# The 100,000 Genomes Pilot on Rare Disease Diagnosis in Healthcare − A Preliminary Report

**DOI:** 10.1056/NEJMoa2035790

**Published:** 2021-11-11

**Authors:** The 100,000 Genomes Project Pilot Investigators, Damian Smedley, Katherine R Smith, Antonio Rueda Martin, Ellen A Thomas, Ellen M McDonagh, Valentina Cipriani, Jamie M Ellingford, Gavin Arno, Arianna Tucci, Jana Vandrovcova, Georgia Chan, Hywel J Williams, Thiloka Ratnaike, Wei Wei, Kathleen Stirrups, Kristina Ibanez, Loukas Moutsianas, Matthias Wielscher, Anna Need, Michael R Barnes, Letizia Vestito, James Buchanan, Sarah Wordsworth, Sofie Ashford, Karola Rehmstrom, Emily Li, Gavin Fuller, Philip Twiss, Olivera Spasic-Boskovic, Sally Halsall, R. Andres Floto, Kenneth Poole, Annette Wagner, Sarju G Mehta, Mark Gurnell, Nigel Burrows, Roger James, Christopher Penkett, Eleanor Dewhurst, Stefan Gräf, Rutendo Mapeta, Mary Kasanicki, Andrea Haworth, Helen Savage, DipRCPath, Melanie Babcock, Martin G Reese, Mark Bale, Emma Baple, Christopher Boustred, Helen Brittain, Anna de Burca, Marta Bleda, Andrew Devereau, Dina Halai, Eik Haraldsdottir, Zerin Hyder, Dalia Kasperaviciute, Christine Patch, Dimitris Polychronopoulos, Angela Matchan, Razvan Sultana, Mina Ryten, Ana Lisa Taylor Tavares, Carolyn Tregidgo, Clare Turnbull, Matthew Welland, Suzanne Wood, Catherine Snow, Eleanor Williams, Sarah Leigh, Rebecca E Foulger, Louise C Daugherty, Olivia Niblock, Ivone U.S. Leong, Caroline F Wright, Jim Davies, Charles Crichton, James Welch, Kerrie Woods, Lara Abulhoul, Paul Aurora, Detlef Bockenhauer, Alexander Broomfield, Maureen A Cleary, Tanya Lam, Mehul Dattani, Emma Footitt, Vijeya Ganesan, Stephanie Grunewald, Sandrine Compeyrot-Lacassagne, Francesco Muntoni, Clarissa Pilkington, Rosaline Quinlivan, Nikhil Thapar, Colin Wallis, Lucy R Wedderburn, Austen Worth, Teofila Bueser, Cecilia Compton, Charu Deshpande, Hiva Fassihi, Eshika Haque, Louise Izatt, Dragana Josifova, Shehla Mohammed, Leema Robert, Sarah Rose, Deborah Ruddy, Robert Sarkany, Genevieve Say, Adam C Shaw, Agata Wolejko, Bishoy Habib, Gavin Burns, Sarah Hunter, Russell J Grocock, Sean J Humphray, Peter N Robinson, Melissa Haendel, Michael A Simpson, Siddharth Banka, Jill Clayton-Smith, Sofia Douzgou, Georgina Hall, Huw B Thomas, Raymond T O’Keefe, Michel Michaelides, Anthony T Moore, Sam Malka, Nikolas Pontikos, Andrew C Browning, Volker Straub, Gráinne S Gorman, Rita Horvath, Richard Quinton, Andrew M Schaefer, Patrick Yu-Wai-Man, Doug M Turnbull, Robert McFarland, Robert W Taylor, OConnor Emer, Yip Janice, Newland Katrina, Huw R Morris, James Polke, Nicholas W Wood, Carolyn Campbell, Carme Camps, Kate Gibson, Nils Koelling, Tracy Lester, Andrea H Németh, Claire Palles, Smita Patel, Noemi BA Roy, Arjune Sen, John Taylor, Pilar Cacheiro, Julius O Jacobsen, Eleanor G Seaby, Val Davison, Lyn Chitty, Angela Douglas, Kikkeri Naresh, Dom McMullan, Sian Ellard, I. Karen Temple, Andrew D Mumford, Gill Wilson, Phil Beales, Maria Bitner-Glindzicz, Graeme Black, John R Bradley, Paul Brennan, John Burn, Patrick F Chinnery, Perry Elliott, Frances Flinter, Henry Houlden, Melita Irving, William Newman, Shamima Rahman, John A Sayer, Jenny C Taylor, Andrew R Webster, Andrew OM Wilkie, Willem H Ouwehand, F Lucy Raymond, NIHR Bioresource, John Chisholm, Sue Hill, David Bentley, Richard H Scott, Tom Fowler, Augusto Rendon, Mark Caulfield

**Affiliations:** 1 Genomics England, Charterhouse Square, London, EC1M 6BQ, United Kingdom; 2 William Harvey Research Institute, Queen Mary University of London, Charterhouse Square, London, EC1M 6BQ, United Kingdom; 3 Open Targets and European Molecular Biology Laboratory - European Bioinformatics Institute (EMBL-EBI), Wellcome Genome Campus, Hinxton, CB10 1SD, United Kingdom; 4 UCL Institute of Ophthalmology, University College London, 11-43 Bath St, London, EC1V 9EL, United Kingdom; 5 Moorfields Eye Hospital NHS Foundation Trust, London, EC1V 2PD, United Kingdom; 6 UCL Genetics Institute, University College London, Gower Street, London, WC1E 6BT, United Kingdom; 7 Division of Evolution and Genomic Sciences, Faculty of Biology, Medicine and Health, University of Manchester, Manchester, M13 9PL, United Kingdom; 8 Manchester Centre for Genomic Medicine, St Mary’s Hospital, Manchester University Foundation NHS Trust, Manchester, M13 9WL, United Kingdom; 9 The National Hospital for Neurology and Neurosurgery, Queen Square, London, WC1N 3BG, United Kingdom; 10 GOSgene, UCL Great Ormond Street Institute of Child Health, London, WC1N 1EH, United Kingdom; 11 Genetic and Genomic medicine, Institute of Medical Genetics, Cardiff University,, Cardiff, CF14 4AY, United Kingdom; 12 Department of Clinical Neurosciences, University of Cambridge, Cambridge Biomedical Campus, Cambridge, CB2 0QQ, United Kingdom; 13 MRC Mitochondrial Biology Unit, University of Cambridge, Cambridge Biomedical Campus, Cambridge, CB2 0XY, United Kingdom; 14 Department of Paediatrics, University of Cambridge, Cambridge Biomedical Campus, Cambridge, CB2 0QQ, United Kingdom; 15 NIHR BioResource, Cambridge University Hospitals, Cambridge Biomedical Campus, Cambridge, CB2 0QQ, United Kingdom; 16 Department of Haematology, University of Cambridge, Cambridge, CB2 0PT, United Kingdom; 17 Great Ormond Street Hospital for Children NHS Foundation Trust, London, WC1N 3JH, United Kingdom; 18 Genetics and Genomic Medicine Programme, UCL Great Ormond Street Institute of Child Health, 30 Guilford St, London, WC1N 1EH, United Kingdom; 19 UCL Ear Institute, London, WC1X 8EE, United Kingdom; 20 Health Economics Research Centre, University of Oxford, Oxford, OX3 7LF, United Kingdom; 21 NIHR Oxford Biomedical Research Centre, Oxford, OX3 9DU, United Kingdom; 22 School of Clinical Medicine, University of Cambridge, Cambridge, CB2 0SP, United Kingdom; 23 Addenbrookes Hospital NHS Trust, Cambridge Biomedical Campus, Cambridge, CB2 0QQ, United Kingdom; 24 Wellcome-MRC Institute of Metabolic Science and NIHR Cambridge Biomedical Research Centre, Cambridge Biomedical Campus, Cambridge, CB2 0QQ, United Kingdom; 25 Department of Medicine, University of Cambridge, Cambridge Biomedical Campus,, Cambridge, CB2 0QQ, United Kingdom; 26 Congenica Ltd, Wellcome Genome Campus, Cambridge, CB10 1DR, United Kingdom; 27 Fabric Genomics, 1611 Telegraph Ave #500, Oakland, CA 94612, U.S.A; 28 University of Exeter Medical School, Exeter, EX2 5DW, United Kingdom; 29 Peninsula Clinical Genetics Service, Royal Devon & Exeter NHS Foundation Trust, Exeter, United Kingdom; 30 Oxford Centre for Genomic Medicine, Oxford University Hospitals NHS Foundation Trust, Oxford, OX3 7LD, United Kingdom; 31 NIHR Great Ormond Street Hospital Biomedical Research Centre,, University College, London, WC1N 3JH, United Kingdom; 32 Clinical Genetics Department, Guy’s & St Thomas’ NHS Foundation Trust, London, SE1 9RT, United Kingdom; 33 Division of Genetics and Epidemiology, Institute of Cancer Research, London, SM2 5NG, United Kingdom; 34 Metabolic Unit, Great Ormond Street Hospital for Children NHS Foundation Trust, London, WC1N 3JH, United Kingdom; 35 Infection, Immunity and Inflammation Research and Teaching Department, UCL Great Ormond Street Institute of Child Health, London, WC1N 1EH, United Kingdom; 36 Department of Renal Medicine, UCL, London, United Kingdom; 37 London Centre for Paediatric Endocrinology and Diabetes, Great Ormond Street Hospital for Children NHS Foundation Trust, London, WC1N 3JH, United Kingdom; 38 NIHR GOSH Biomedical Research Centre, London, WC1N 3JH, United Kingdom; 39 Department of Gastroenterology, Great Ormond Street Hospital for Children NHS Foundation Trust, London, WC1N 3JH, United Kingdom; 40 Stem cells and Regenerative Medicine, UCL Great Ormond Street Institute of Child Health, London, WC1N 1EH, United Kingdom; 41 Florence Nightingale Faculty of Nursing, Midwifery & Palliative Care, King’s College London, London, SE1 8WA, United Kingdom; 42 St John’s Institute of Dermatology, Guy’s & St Thomas’ NHS Foundation Trust, London, SE1 9RT, United Kingdom; 43 Illumina Cambridge Ltd, Cambridge, CB21 6DF, United Kingdom; 44 The Jackson Laboratory for Genomic Medicine, 10 Discovery Drive, Farmington, CT, 06032, USA; 45 Center for Genome Research and Biocomputing, Environmental and Molecular Toxicology, Oregon State University,, Corvallis, OR, 97331, USA; 46 Division of Genetics and Molecular Medicine, King’s College London, London, SE1 9RT, United Kingdom; 47 Ophthalmology Department, UCSF School of Medicine, San Francisco, CA, 94143-0644, USA; 48 Newcastle Eye Centre, Royal Victoria Infirmary, Newcastle upon Tyne, NE1 4LP, United Kingdom; 49 Institute of Genetic Medicine, Newcastle University, International Centre for Life, Newcastle upon Tyne, United Kingdom; 50 Wellcome Centre for Mitochondrial Research, Translational and Clinical Research Institute, Faculty of Medical Sciences, Newcastle University, Newcastle upon Tyne, NE2 4HH, United Kingdom; 51 Highly Specialised Mitochondrial Service, Newcastle upon Tyne Hospitals NHS Foundation Trust, Newcastle upon Tyne, NE1 4LP, United Kingdom; 52 NIHR Newcastle Biomedical Research Centre, Newcastle upon Tyne, NE4 5PL, United Kingdom; 53 Translational and Clinical Research Institute, Faculty of Medical Sciences, Newcastle University, Central Parkway, Newcastle upon Tyne, NE1 3BZ, United Kingdom; 54 Newcastle upon Tyne Hospitals NHS Foundation Trust, Newcastle upon Tyne, NE7 7DN, United Kingdom; 55 Cambridge Centre for Brain Repair, Department of Clinical Neurosciences, University of Cambridge, Cambridge, CB2 0PY, United Kingdom; 56 NIHR Biomedical Research Centre at Moorfields Eye Hospital and UCL Institute of Ophthalmology, London, EC1V 2PD, United Kingdom; 57 Oxford Genetics Laboratories, Oxford University Hospitals NHS Foundation Trust, The Churchill Hospital, Oxford, OX3 7LE, United Kingdom; 58 Wellcome Centre for Human Genetics, University of Oxford, Oxford, OX3 7BN, United Kingdom; 59 MRC Weatherall Institute of Molecular Medicine, University of Oxford, John Radcliffe Hospital, Oxford, OX3 9DS, United Kingdom; 60 Nuffield Department of Clinical Neurosciences, Level 6, West Wing, John Radcliffe Hospital, Oxford, OX3 9DU, United Kingdom; 61 Institute of Cancer and Genomic Sciences, Institute of Biomedical Research, University of Birmingham, Edgbaston campus, Birmingham, B15 2TT, United Kingdom; 62 Department of Clinical Immunology, John Radcliffe Hospital, Oxford, OX3 9DU, United Kingdom; 63 Department of Haematology, Oxford University Hospital Foundation Trust, Oxford, OX3 9DU, United Kingdom; 64 Oxford Epilepsy Research Group, Nuffield Department of Clinical Neurosciences, University of Oxford, John Radcliffe Hospital, Oxford, OX3 9DU, United Kingdom; 65 Department of Neurology, Oxford University Hospitals NHS Foundation Trust, Oxford, OX3 9DU, United Kingdom; 66 Genomic Informatics Group, University Hospital Southampton, Southampton, SO16 6YD, United Kingdom; 67 NHS England and NHS Improvement, Skipton House, 22 London Rd, Elephant and Castle, London, SE1 6JW, United Kingdom; 68 Liverpool Women’s NHS Foundation Trust, Liverpool, L8 7SS, United Kingdom; 69 Imperial College Healthcare NHS Trust, Hammersmith Hospital, London, W12 0HS, United Kingdom; 70 Birmingham Women’s Hospital, Birmingham, B15 2TG, United Kingdom; 71 University of Exeter Medical School, Royal Devon and Exeter Hospital, Exeter, EX2 5DW, United Kingdom; 72 University of Southampton, Southampton, SO17 1BJ, United Kingdom; 73 University Hospital Southampton, Southampton, SO16 6YD, United Kingdom; 74 School of Cellular and Molecular Medicine, University of Bristol, Bristol, BS2 8HW, United Kingdom; 75 Yorkshire and Humber, Sheffield Children’s Hospital, Sheffield, S10 2TH, United Kingdom; 76 Northern Genetics Service, Newcastle upon Tyne Hospitals NHS Foundation Trust, Newcastle upon Tyne, NE1 3BZ, United Kingdom; 77 Institute of Cardiovascular Science, University College London, Gower Street, London, WC1E 6BT, United Kingdom; 78 Division of Medical and Molecular Genetics, 8th Floor Tower Wing, King’s College London, London, SE1 9RT, United Kingdom; 79 Mitochondrial Research Group, UCL Great Ormond Street Institute of Child Health, London, WC1N 3JH, United Kingdom; 80 NIHR Newcastle Biomedical Research Centre, Newcastle upon Tyne, NE45PL, United Kingdom; 81 NHS Blood and Transplant, Cambridge Biomedical Campus, Cambridge, United Kingdom; 82 Wellcome Sanger Institute, Wellcome Genome Campus, Hinxton, Cambridge, United Kingdom

## Abstract

**Background:**

The UK 100,000 Genomes Project is in the process of investigating the role of genome sequencing of patients with undiagnosed rare disease following usual care, and the alignment of research with healthcare implementation in the UK’s national health service. (Other parts of this Project focus on patients with cancer and infection.)

**Methods:**

We enrolled participants, collected clinical features with human phenotype ontology terms, undertook genome sequencing and applied automated variant prioritization based on virtual gene panels (PanelApp) and phenotypes (Exomiser), alongside identification of novel pathogenic variants through research analysis. We report results on a pilot study of 4660 participants from 2183 families with 161 disorders covering a broad spectrum of rare disease.

**Results:**

Diagnostic yields varied by family structure and were highest in trios and larger pedigrees. Likely monogenic disorders had much higher diagnostic yields (35%) with intellectual disability, hearing and vision disorders, achieving yields between 40 and 55%. Those with more complex etiologies had an overall 25% yield. Combining research and automated approaches was critical to 14% of diagnoses in which we found etiologic non-coding, structural and mitochondrial genome variants and coding variants poorly covered by exome sequencing. Cohort-wide burden testing across 57,000 genomes enabled discovery of 3 new disease genes and 19 novel associations. Of the genetic diagnoses that we made, 24% had immediate ramifications for the clinical decision-making for the patient or their relatives.

**Conclusion:**

Our pilot study of genome sequencing in a national health care system demonstrates diagnostic uplift across a range of rare diseases.

(Funded by National Institute for Health Research and others)

Rare disease is a worldwide healthcare challenge with approximately 10,000 disorders affecting 6% of the population in Western societies.^1,2^ Over 80% of rare diseases have a genetic component and these conditions are disabling and expensive to manage. One-third of children with a rare disease die before their fifth birthday.^1^ The adoption of next generation sequencing has improved rare disease diagnostic rates over the past decade.^3–5^ However, the majority of rare disease patients remain without a molecular diagnosis following standard diagnostic testing.^3–5^ To address this, the UK Government launched the 100,000 Genomes Project (100KGP) in 2013 to apply whole genome sequencing (WGS) to rare disease, cancer and infection in national healthcare.^6^

To assess impact of this WGS approach on the genetic diagnosis of rare disease in the UK’s National Health Service, we carried out a pilot study in which we enrolled families and undertook detailed clinical phenotyping of the proband.^4^ We collected electronic health records from all participants in a multi-petabyte research environment.^5^ When necessary, we carried out wet bench orthogonal tests and *in-silico* approaches.

## Methods

### Patients

Following ethical approval, consenting participants (identified by healthcare professionals and researchers) with a broad range of rare diseases without diagnoses after undergoing usual care in the NHS (which ranged from no available test through approved tests which did not include genome sequencing) were recruited by nine English hospitals and consented through the National Institute for Health Research (NIHR) BioResource for Rare Diseases. To test the broad applicability of genome sequencing, participants were eligible if they had a rare disease (as defined in the UK as a disorder affecting 1 in 2000 or less), were likely to have a single gene or oligogenic aetiology, and no genomic diagnosis. Data on prior proband testing was collected where possible including single-gene tests, karyotyping, single nucleotide polymorphism (SNP) arrays, next generation sequencing panels, and exomes. Probands and, where feasible, parents and/or other family members were enrolled by multiple clinical specialties in the NHS. Standardized baseline clinical data were recorded using the Human Phenotype Ontology (HPO)^7^ against disease specific data models^8^ and whole blood was drawn for DNA extraction. The participants are followed over their life course using electronic health records (all hospital episodes, registries and cause of death).

### Genome Sequencing

Genome sequencing^9^ was performed using the Illumina TruSeq DNA PCR-Free sample preparation kit by Illumina Laboratory Sciences, Cambridge UK on an HiSeq 2500 sequencer, generating a mean depth of 32× (range from 27× to 54×) and greater than 15× for at least 95% of the reference human genome. WGS reads were aligned to the Genome Reference Consortium human genome build 37 (GRCh37) using Isaac Genome Alignment Software. Family-based variant calling of single variant nucleotides and insertion deletions (indels) for chromosomes 1 to 22, X, and the mitochondrial genome (mean 2814x coverage, range 142-16581) was performed using the Platypus variant caller.^10^

### The Diagnostic Pipeline

We constructed an automated analytical pipeline to filter the genome down to rare, segregating and predicted damaging candidate variants in coding regions. To limit the possibility of overlooking, or inefficiently prioritizing diagnoses we focussed initially on virtual gene panels based on both the recruited clinical indication/disease and submitted HPO terms (applied virtual panels). To address the issue of which genes have sufficient evidence to attribute causation and include in these virtual gene panels, we used our PanelApp software to enable expert, crowd-sourced review and curation of genes with diagnostic-grade evidence for each of our disease categories e.g. evidence in at least three, unrelated families.^11^ Loss of function (LoF) or *de novo*, protein altering variants affecting genes in the applied virtual panels were classified as tier 1, other variant types such as missense variants affecting these genes were classified as tier 2, and all other filtered variants were classified as tier 3 (Figure S1 in the Supplementary Appendix). To further reduce the possibility of missing, or inefficient prioritization of diagnoses, we ran Exomiser^12^, a phenotype-based approach to look across all genes in the genome for a diagnosis. Exomiser prioritizes rare, segregating, predicted pathogenic variants in genes where the patient phenotypes match previous reference knowledge from human disease or model organism databases. The ontology-driven phenotype matching can detect patients possessing atypical profile for a disease.

Decision support systems and clinical genetics teams provided by Congenica Ltd and Fabric Genomics^13,14^ assisted us in variant prioritization and return of candidate variants to the 13 NHS Genomic Medicine Centres (GMC). These variants were reviewed by NHS clinical scientists and clinicians using the American College of Medical Genetics and Genomics guidelines and a diagnostic report was issued for each proband.^15^ Final clinical outcomes included whether a genetic diagnosis was obtained, the variant(s) involved, whether they explained all, or some of the phenotypes and whether an intervention was deployed.

The pilot participants were recruited and sequenced throughout 2014-2016, while the infrastructure to collect, QC, process and return data was being established. Results were returned to the GMCs from May 2016 to April 2019. In our post-pilot phase with an established pipeline, we now return results to the GMCs within 6 weeks of sample collection.

### Novel Pathogenic Variants

Researchers investigated coding and non-coding regions for novel diagnoses in genes matching the patients’ phenotypes, including the presence of *de novo* variants in highly constrained coding regions^16^ with 95% confidence. We used a novel methodology for mitochondrial DNA that accounts for heteroplasmy,^17^ Genomiser,^18^ and ExpansionHunter for simple tandem repeat expansions.^19^ Finally we employed a novel random forest method to analyse Canvas^20^ and Manta^21^ calls and identify potentially pathogenic copy number and structural variants.

Gene-based burden testing to detect enrichment of rare, predicted pathogenic, segregating variants in novel genes in specific disease cohorts relative to controls was performed on the pilot genomes as well as additional genomes from the rest of the 100KGP to increase power (57,002 genomes; see Supplementary Methods).

Access to the pilot genomic and clinical data is freely accessible by becoming a member of a Genomics England Clinical Interpretation Partnership (GeCIP) domain (https://www.genomicsengland.co.uk/about-gecip/).

### Statistical Analysis

Testing was performed using the R (version 3.6.0) and Stata (version 16) statistical packages. Further detail on individual methods is given in the Supplementary Appendix.

## Results

### Patients

We enrolled 4660 participants (2183 probands and 2477 family members) from 161 broad categories across rare disease (Table 1), with neurologic, ophthalmologic and tumor syndromes commonly represented. Participants were recruited with varying numbers of affected and unaffected family members. We aimed, with varying degrees of success, to recruit trios or larger family structures to facilitate more effective variant prioritization. Of the probands with multiple bowel polyps whom we recruited, 93% were singletons. In contrast, 12% of probands with intellectual disability were singletons. Adult probands were more commonly enrolled than pediatric probands (age at recruitment 18 years or younger) (74% vs. 26%), in line with the general population (79% vs. 21%; 2011 census of England and Wales). The preponderance of adults is unusual compared to previous sequencing projects and reflects an eligibility criterion: probands had already undergone usual care: in many cases, usual care involved standard genetic testing (mostly single-gene or panel-based). A lower percentage of female probands were recruited, especially for pediatric cases, where the difference was significant (232 female vs. 339 male; *P<* 0.001) based on the expected female proportion of 51% from 2011 census of England and Wales) across most disease categories. The increased susceptibility of males to recessive X-linked conditions may account for this sex bias: over 6% of total diagnoses involved variants on the X chromosome (which represents approximately 5% of the genome). The inferred ancestry of the probands (see Supplementary Appendix) was in line with that expected from the population (86% white, 7.5% Asian, 3.3% black, 2.2% mixed, 1% other: 2011 census of England and Wales). However, significantly more pediatric probands were of South Asian ancestry compared to adult probands (16% vs. 4%, P<0.001); our results indicated potential consanguinity in 43% of pediatric South Asian probands and 1% for the other pediatric probands (Table 1).

### Clinical Data and Sequencing

We collected HPO terms for each participant (median of 4 present terms, range 1-61 and median of 4 absent terms (phenotypes not exhibited by the proband), range 0-144). We then carried out genome sequencing followed by quality assurance to check coverage, sequence quality, presence of repeat sample submissions or sample swaps, and consistency with reported family structures (see Supplementary Appendix).

### The Diagnostic Yield

**Figure 1:**
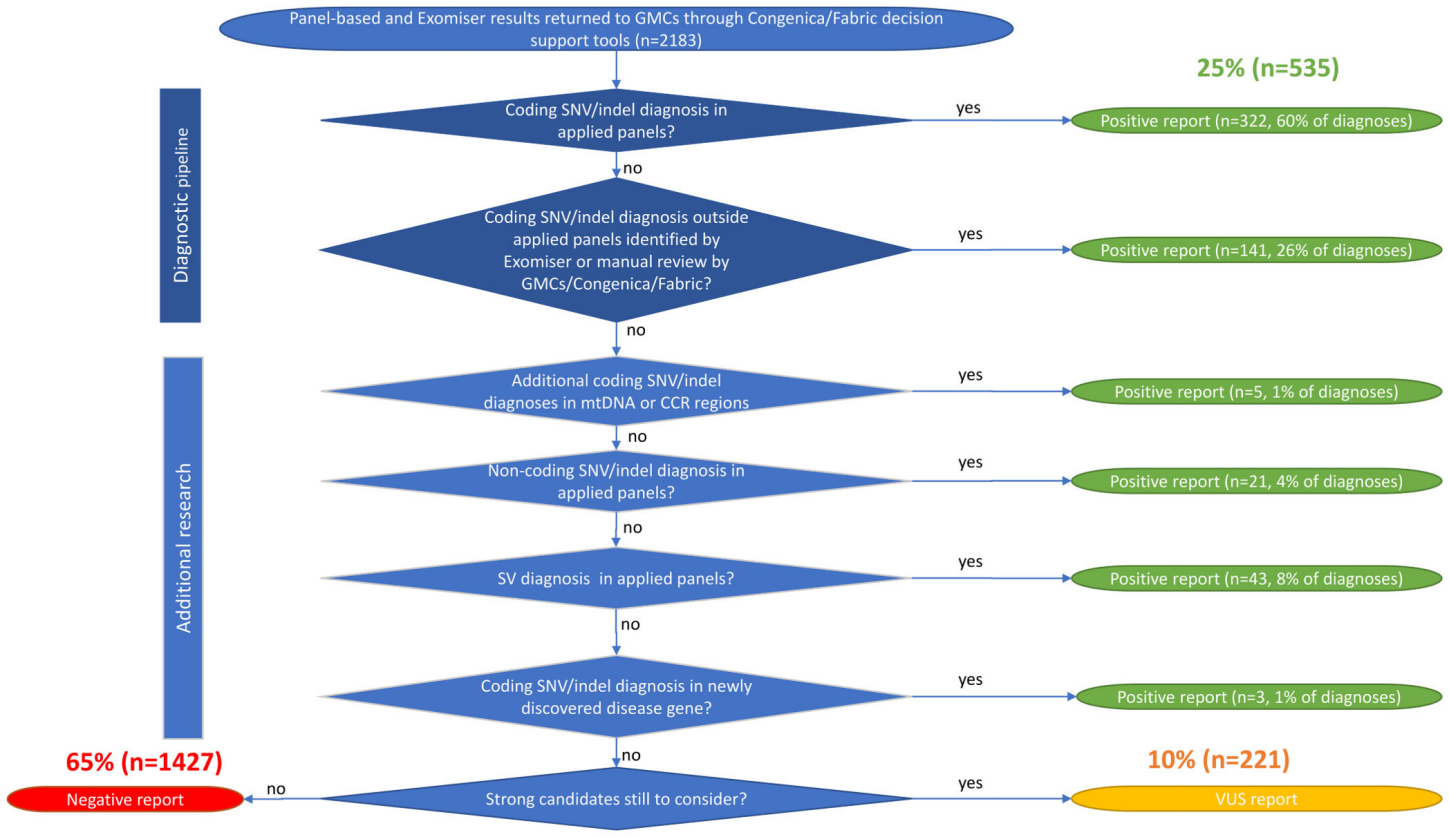
Overview of the diagnostic and research pipeline and source of diagnoses. Results were returned to the Genomic Medicine Centres (GMCs) of the recruiting hospitals on an 2183 pilot probands. 25% received a positive diagnosis, 10% had variant(s) of unknown significance (VUS) in genes consistent with the phenotype according to clinical geneticists at the recruiting site, but with further functional validation required. The remaining 65% received a negative report at the time but will be reanalysed. Numbers and source of these positive diagnoses is shown at each stage of the automated diagnostic pipeline and additional research where a clear diagnosis was not immediately obvious.

**Figure 2:**
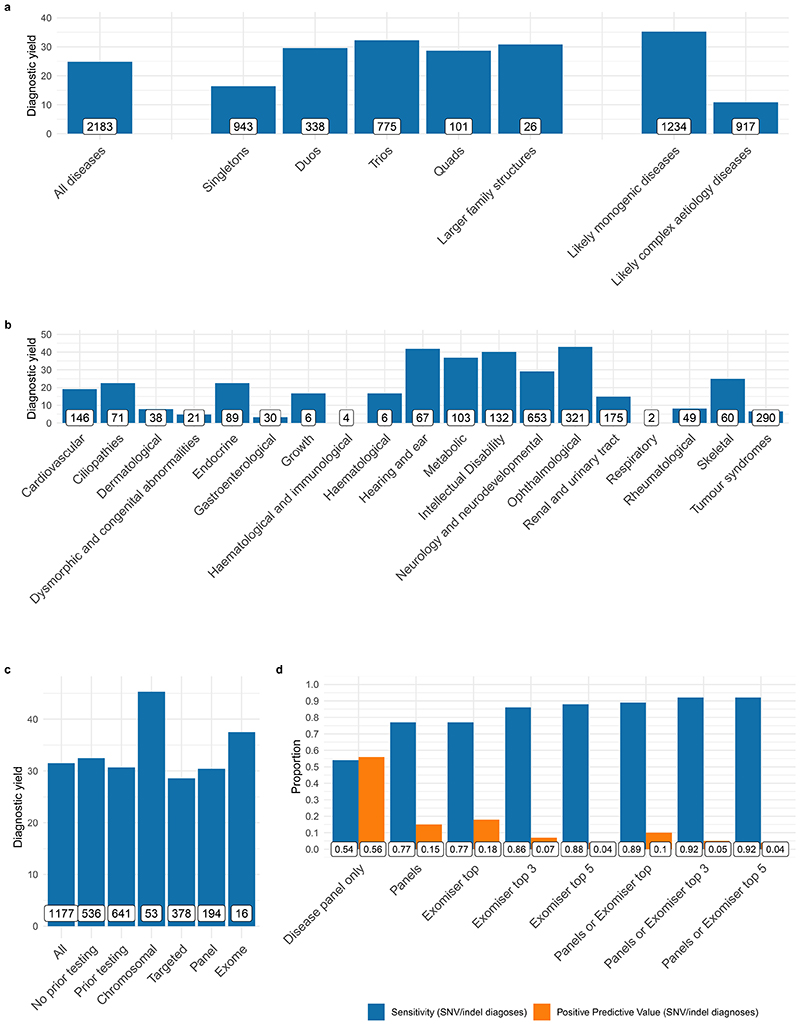
Diagnoses in the rare disease pilot. (a) Percentage diagnostic yield for all samples and sub-divided by family structure or whether likely monogenic (35% yield) vs more complex aetiologies (11% yield) with the numbers of probands shown on bars, (b) Percentage diagnostic yield by disease area (numbers of closed probands shown on bars), (c) Percentage diagnostic yield for probands with/without prior genetics testing and broken down by most extensive testing type: chromosomal (karyotyping, arrayCGH, SNP arrays), targeted single gene tests, NGS panels or WES (numbers of closed probands shown on bars) (d) Performance of virtual panel-based and Exomiser prioritization for identifying the diagnoses. Virtual disease panel only: a single panel for the recruited disease category. Applied panels - all applied virtual panels used in the pipeline including the recruited disease associated panel as well as 0 or more additionally selected panels based on the patient phenotypes (HPO terms). Proportion of diagnoses detected are in blue (sensitivity) along with proportion of prioritized variants leading to a positive diagnosis in orange (positive predictive value). Proportions are also shown on bars. Here, diagnosed variant(s) are true positives and other returned candidate variants are false positives.Table 1. Demographics (including inferred ancestry) of the 100,000 Genomes Project pilot.

We obtained genetic diagnoses for 25% of probands and deposited the genotypes into the ClinVar repository (accession numbers XXXX to YYYY). Of these diagnoses, 60% were made on the basis of coding SNV/indels in the applied virtual panels, 26% from coding SNV/indels affecting well-established disease genes outside the virtual panels using phenotype-based prioritization and/or expert review by the clinicians, Congenica Ltd, or Fabric Genomics, and 14% from genome-wide, phenotype-agnostic research analysis looking beyond SNV/indels, coding regions, and disease genes in the virtual panels (Figure 1). Following international guidelines^15^ a further 10% of probands were classified with variants of unknown significance in genes consistent with the phenotype by clinical review at the site, but with further functional validation required. Fewer candidate variants were returned after filtering in larger family structures (Table 3), making it easier to identify causative variants, in turn leading to higher diagnostic rates for trios, quads and more complex family structures (Figure 2a), even within a disorder e.g. for hereditary ataxia the diagnostic rate increased from 21% for singletons to 32% for trios (Table S4 in the Supplementary Materials).

Unsurprisingly, we obtained a higher diagnostic yield for diseases that were considered more likely to have a monogenic cause (Table S4 in the Supplementary Appendix) than those we considered more likely to have complex etiology (35% vs 11%) (Figure 2a). Likely monogenic diseases equate to those with a presence in OMIM and where genetic testing is part of the standard diagnostic workup, based on the consensus blinded review of three clinical geneticists. Diagnostic yield was highly variable by disease (Figure 2b, Table S3 in the Supplementary Appendix), varying from 40-55% for intellectual disability and various vision and hearing disorders to 6% for tumor syndromes.

We obtained data on the presence or absence of prior genetic testing for a subset (1177) of the participants. The number of tests per proband ranged from 0-16 with a median of 1 (IQR 0-2), and approximately half of the probands in this subset had been tested at least once. The overall diagnostic uplift from genome sequencing in this subset was 32% with only a slight difference depending on whether prior testing had been performed (33%), or not (31%). However, many of these prior tests were not recent. The diagnostic yield provided by genome sequencing varied between 28 to 45% depending on the type of prior testing (Figure 2c, Table S5 in the Supplementary Appendix) which, for the most part, involved targeted single gene and panel testing (Table S6 in the Supplementary Appendix).

### Diagnostic Pipeline

The aim of the automated, diagnostic pipeline is to identify a few, potentially causative candidate variants, from the millions in a whole genome, through removal of extremely unlikely candidates (filtering) and identification of the most likely in the remainder (prioritization). This allows the GMCs to efficiently perform manual, clinical interpretation and issue a diagnostic report. The virtual panel-based pipeline identified 322 (66%) of the 490 SNV/indel-based diagnoses from the genomes, with a high positive predictive value given the millions of variants in the whole genomes: of 1041 of returned candidate variants, 291 (28%) proved to be diagnostic. We re-ran this analysis in December 2019 to assess the impact of using updated versions of the virtual panels containing the latest disease gene discoveries, improved virtual panel selection based on the patient’s phenotype and advances in variant filtering strategies, e.g. allowing for incomplete penetrance where suspected. This increased the number of genetic diagnoses detected from 322 to 377 (77%) with a positive predictive value of 15% (Figure 2d), demonstrating effective filtering and prioritization of the variants with only a median of 1 (IQR 0-2) candidate variant in panels returned to the clinicians at the GMCs per case (Table 3). Ongoing evolution of the virtual panels with new disease genes is expected to continue increasing the yield from this approach.

Phenotype-based prioritization using Exomiser detected 77%, 86%, and 88% of these diagnoses in the top, top 3 and top 5 ranked candidates respectively (Figure 2d). Exomiser and use of virtual panels were complementary, with 92% of these diagnoses re-called when used combined (last blue bar in Figure 2d). Precision phenotyping of our patients was essential both for Exomiser and for the selection of additional virtual panels, without which only 54% of these diagnoses would have been prioritized in the recruited disease virtual panel and presented to the GMCs as a likely candidate (first blue bar in Figure 2d).

### Research-based Diagnoses

14% of the genetic diagnoses required research outside the diagnostic pipeline (Figure 1). This research involved comparisons with the genome sequences and clinical data in our research environment, with validation using wet bench orthogonal tests and *in-silico* approaches (Table S7 in the Supplementary Appendix). Additional diagnoses were made by screening for the presence of *de novo* variants in highly constrained coding regions^16^. These diagnoses included a de novo *EBF3* missense variant in a patient with hereditary ataxia. Mitochondrial genome analysis, taking into account heteroplasmy, detected 4 new diagnoses as well as the 9 that had already been detected by the main pipeline). Twelve probands had intronic splicing variants prioritized by Exomiser due to the known pathogenic status of these variants in ClinVar.^23^ Nine novel non-coding diagnoses involving previously undescribed variants required exploration of the whole genome and *in vitro* functional validation via reverse transcription polymerase chain reaction, mini-gene, or luciferase assays.^24,25,26^ Here, unsolved probands were queried for non-coding variants affecting genes in the applied virtual panels, either alone, or in compound heterozygosity with loss-of-function variants. These were identified using either Genomiser or, for retinal disorder probands, systematic analysis of the untranslated regions, promoter or introns. A further 43 probands were fully or partially explained by structural variants or simple tandem repeat expansions in the genes *HTT* or *FXN* in probands with hereditary spastic paraplegia.

### Novel Disease Gene Associations

We performed burden testing to discover novel Mendelian disease gene associations and potential genetic diagnoses for unsolved probands; 828 significant disease-gene associations (q value < 0.1) were identified, including 249 known and 579 novel genes (novel with respect to their association with disease), with only 0.03 ± 0.2 (range 0-3) associations from 10,000 permutations where cases and controls were assigned randomly. Twenty two candidates represent the most likely new, fully penetrant, Mendelian disease genes (Table S8 in the Supplementary Appendix and ClinVar accession numbers SCV001759972 - SCV001760540) with three recently independently confirmed diagnoses: *UBAP1* in hereditary spastic paraplegia,^27^ *FOXJ1* in non-CF bronchiectasis,^28^ and *SORD* in Charcot-Marie Tooth disease.^29^ Diagnostic reports were issued for three probands with these genes (Figure 1) and we are investigating others in GeneMatcher and by functional validation studies in model organisms.

### Diagnostic Sequelae

These findings ended long diagnostic odysseys for some patients and their families (the median duration of odyssey was 75 months and number of hospital visits was 68); Table S1 in Supplementary Appendix); we speculate that they will mitigate NHS resource costs (183,273 episodes of hospital care costing £87 million for affected participants; Table S3 in Supplementary Appendix). In addition, 134 (25%) of the 533 genetic diagnoses were reported by clinicians to be of immediate clinical actionability with only 11 (0.2%) described as having no benefit. As of now, the remainder of the diagnoses are of unknown utility. Healthcare benefits included 4 diagnoses leading to a suggested change in medication, 26 suggesting additional surveillance for the proband or relatives, 13 allowing clinical trial eligibility, 59 informing future reproductive choices, and 32 with other benefits (Table S9 in the Supplementary Appendix).

In several specific probands, diagnoses have had important clinical actionability. In a 36-yr-old male with suspected choroideraemia, we detected a novel, *CHM* promoter variant causing loss of gene expression^26^ and offering eligibility for a gene-replacement trial. A male neonate proband presented with severe infection and transient neurologic symptoms immediately after birth and died at 4 months with no diagnosis but healthcare costs of approximately £80,000 (Table S10 in Supplementary Appendix). A diagnosis of transcobalamin 2 deficiency due to a homozygous frameshift in *TCN2* was made from this study which enabled predictive testing to be offered to the younger brother within one week of birth. The younger child, who received a positive result, received weekly hydroxocobalamin injections to prevent metabolic decompensation. A 10-year-old girl was admitted to intensive care with life-threatening chicken pox. She had endured a diagnostic odyssey over seven years at a total cost of £356,571 across 307 secondary care episodes (Table S11 in Supplementary Appendix). We were able to diagnose *CTPS1* deficiency due to a homozygous, known pathogenic splice acceptor variant. A diagnosis enabled a curative bone marrow transplant (cost £70,000) and predictive testing of her siblings showed no further family members to be at risk. One proband had waited till his sixth decade for a genomic diagnosis of an *INF2* mutation causing focal segmental glomerulosclerosis. His father, brother and uncle had all died of renal failue. He had received two kidney transplants, had transmitted the condition to his daughter and was concerned about whether his 15-year-old grand daughter, who was under surveillance, was at risk. After he received his genetic diagnosis, the grand-daughter was tested, found to be negative, and discharged from regular medical surveillance.

## Discussion

Our findings demonstrate a substantial uplift in genomic diagnoses achieved for patients by genome sequencing across a broad spectrum of rare disease. The enhanced diagnostic benefit was observed regardless of whether participants had undergone prior genetic testing (31% in those who had received testing and 33% in those who had not). For 25% of those who received a genetic diagnosis, there was immediate clinical actionability. Standardizing procedures, from enrolment of patients to the return of NHS-validated results to clinicians, was critical to our success. For example, clinical data collection using diseasespecific data models and HPO terms enabled diagnoses confirming the value of standardization through ontologies and clinical annotation in precision medicine.^30^. These additional diagnoses, beyond the 264 (49% of total diagnoses) observed in the single disease virtual panel, came from Exomiser and additional, applied virtual panels. The diagnostic discoveries derived by combining research, decision support and clinical validation and assessment leveraged an additional 72 diagnoses.

Diagnostic yield was influenced by family structure, and for disorders with a likely Mendelian inheritance and a single gene etiology our yield increased to 35%: ophthalmological, metabolic and neurologic disorders yielded the greatest percentage of diagnoses. The scale of our dataset enabled cohort-wide burden testing which identified numerous novel disease–gene associations including three that have now been confirmed and 19 with compelling evidence that are likely to be confirmed in independent datasets.

Of the diseases we diagnosed through genome-sequencing, 13% were caused by mutations in non-coding sequence or mitochondrial genomes, tandem repeat expansions in Huntington disease, and a wide range of structural variants with nucleotide resolution of breakpoints using a novel random forest method. An additional 2% of diagnoses involved coding variants in regions of low coverage on exome sequencing. Our results provide new evidence of the value of genome sequencing and mirror previous studies where 53% of participants who received new diagnoses from genome sequencing had previously received testing by exome sequencing.^5^

Previous studies have demonstrated how next-generation sequencing can reveal diagnoses with yields of between 25% and 29% from exome sequencing in persons who had received no prior genetic testing.^32–34^ The Undiagnosed Disease Network reported a 26% yield from a mixture of exome and genome sequence analysis of 382 patients^5^ and another genome sequencing study gave a 42% yield in 50 families with intellectual disability in whom prior testing had previously been carried out.^35^ We obtained similar results with a broad range of disorders (160) with unmet diagnostic need. Our approach is limited to diagnoses that are readily made through short-read genome sequencing. Fully phased, long-read sequencing better detects structural variation and delivers sequence from parts of the genome that are poorly captured by short read sequencing.^31^

This pilot has underpinned the case for genome-sequencing in the diagnosis of certain specific rare diseases in the new NHS National Genomic Test Directory^36^. For patients in the National Health Service for specific disorders, such as intellectual disability, genome-sequencing will now be the first-line test (Table S12 in the Supplementary Appendix) and the NHS in England, through a new National Genomic Medicine Service, is in the process of sequencing 500,000 whole genomes in rare disease and cancer in healthcare. We hope our findings will assist other health systems in considering the role of genome sequencing in the care of patients with rare diseases.

Disclosure forms provided by the authors are available with the full text of this article at NEJM.org.

## Supplementary Material

Supplement

## Figures and Tables

**Table 1:** Demographics (including inferred ancestry) of the 100,000 Genomes Project pilot.

Variable	All probands (N=2183)	Paediatric (age at recruitment <=18) probands (N=571)	Adult (age at recruitment > 18) probands (N=1612)
**Sex — no. (%)**
**Male**	1138 (52)	339 (16)	799 (37)
**Female**	1045 (48)	232 (11)	813 (37)
	2183 (100)	571 (26)	1612 (74)
**Median (IQR) age in years at recruitment**	35 (18-54)	9 (5-14)	45 (31-60)
Race or ethnic group — no. (%), %consangunity suggested in record			
**African**	50 (2), 0	25 (4), 0	25 (2), 0
**Ad Mixed American**	26 (1), 23	12 (2), 25	14 (1), 21
**East Asian**	8 (<1), 0	2 (<1), 0	6 (<1), 0
**European**	1931 (88), <1	438 (77), <1	1493 (93), <1
**South Asian**	163 (7), 36	93 (16), 43	70 (4), 25
**Not determined**	5 (<1), 0	1 (<1), 0	4 (<1), 0
	2183 (100), 3	571 (26), 8	1612 (74), 2

**Table 2:** Clinical features of the 100,000 Genomes Project pilot

Primary symptoms — no. (%)	All Families	Singletons	Duos	Trios	Larger families
**Cardiovascular**	147 (7)	56 (3)	24 (1)	49 (2)	18 (1)
**Ciliopathies**	69 (3)	34 (2)	14 (1)	16 (1)	5 (<1)
**Dermatological**	38 (2)	9 (<1)	5 (<1)	22 (1)	2 (<1)
**Dysmorphic and congenital abnormalities**	20 (1)	10 (<1)	2 (<1)	7 (<1)	1 (<1)
**Endocrine**	87 (4)	57 (3)	14 (1)	12 (1)	4 (<1)
**Gastroenterological**	32 (1)			18 (1)	14 (1)
**Growth**	3 (<1)	3 (<1)			
**Haematological and immunological**	5 (<1)	2 (<1)	3 (<1)		
**Haematological**	7 (<1)		3 (<1)	2 (<1)	2 (<1)
**Hearing and ear**	35 (2)	6 (<1)	5 (<1)	17 (1)	7 (<1)
**Metabolic**	93 (4)	24 (1)	12 (1)	48 (2)	9 (<1)
**Intellectual disability (ID)**	130 (6)	10 (<1)	24 (1)	78 (4)	18 (1)
**Neurology and neurodevelopmental (excl. ID)**	521 (24)	193 (9)	93 (4)	194 (9)	41 (2)
**Ophthalmological**	348 (16)	74 (3)	62 (3)	199 (9)	13 (1)
**Renal and urinary tract**	176 (8)	125 (6)	21 (1)	26 (1)	4 (<1)
**Respiratory**	2 (<1)	1 (<1)		1 (<1)	
**Rheumatological**	48 (2)	14 (1)	6 (<1)	25 (1)	3 (<1)
**Skeletal**	62 (3)	15 (1)	11 (1)	23 (1)	13 (1)
**Tumour syndromes**	293 (13)	231 (11)	31 (1)	27 (1)	4 (<1)
**Other**	67 (3)	17 (1)	12 (1)	34 (2)	4 (<1)
	2183(100)	881 (40)	343 (16)	797 (37)	162 (7)

**Table 3:** Number of candidate variants returned to the NHS per case by automated virtual panel-based analysis pipeline. Duos refer strictly to parent-child pairs and trios to both parents and a child in a family. Values shown are median (IQR).

	All family structures	Singletons	Duos	Trios	Other family structures
**Variants after filtering**	221 (49-288)	292 (258-327)	149 (117-213)	29 (17-136)	22.5 (9-71)
**In virtual panels**	1 (0-2)	1 (0-2)	1 (0-3)	1 (0-2)	0 (0-1)

## References

[1] Annual Report of the Chief Medical Officer 2016 Generation Genome.

[2] Ferreira CR (2019). The burden of rare diseases. Am J Med Genet A.

[3] Boycott KM, Rath A, Chong JX (2017). International Cooperation to Enable the Diagnosis of All Rare Genetic Diseases. Am J Hum Genet.

[4] Taylor JC, Martin HC, Lise S (2015). Factors influencing success of clinical genome sequencing across a broad spectrum of disorders. Nat Genet.

[5] Splinter K, Adams DR, Bacino CA (2018). Undiagnosed Diseases Network. Effect of Genetic Diagnosis on Patients with Previously Undiagnosed Disease. N Engl J Med.

[6] The Genomics England Protocol 2017.

[7] Köhler S, Carmody L, Vasilevsky N (2019). Expansion of the Human Phenotype Ontology (HPO) knowledge base and resources. Nucleic Acids Res.

[8] Genomics England data models 2018.

[9] Bentley DR, Balasubramanian S, Swerdlow HP (2008). Accurate whole human genome sequencing using reversible terminator chemistry. Nature.

[10] Rimmer A, Phan H, Mathieson I, Iqbal Z (2014). Integrating mapping-, assembly- and haplotype-based approaches for calling variants in clinical sequencing applications. Nat Genet.

[11] Martin AR, Williams E, Foulger RE (2019). PanelApp crowdsources expert knowledge to establish consensus diagnostic gene panels. Nat Genet.

[12] Smedley D, Jacobsen JO, Jäger M (2015). Next-generation diagnostics and disease-gene discovery with the Exomiser. Nat Protoc.

[13] Congenica platform.

[14] Fabric Genomics platform.

[15] Richards S, Aziz N, Bale S, Bick D, ACMG Laboratory Quality Assurance Committee (2015). Standards and guidelines for the interpretation of sequence variants: a joint consensus recommendation of the American College of Medical Genetics and Genomics and the Association for Molecular Pathology. Genet Med.

[16] Havrilla JM, Pedersen BS, Layer RM, Quinlan AR (2019). A map of constrained coding regions in the human genome. Nat Genet.

[17] Wei W, Tuna S, Keogh MJ, Smith KR (2019). Germline selection shapes human mitochondrial DNA diversity. Science.

[18] Smedley D, Schubach M, Jacobsen JOB (2016). A Whole-Genome Analysis Framework for Effective Identification of Pathogenic Regulatory Variants in Mendelian Disease. Am J Hum Genet.

[19] Dolzhenko E, van Vugt JJFA, Shaw RJ (2017). Detection of long repeat expansions from PCR-free whole-genome sequence data. Genome Res.

[20] Zhang L, Bai W, Yuan N, Du Z (2019). Comprehensively benchmarking applications for detecting copy number variation. PLoS Comput Biol.

[21] Kosugi S, Momozawa Y, Liu X, Terao C, Kubo M, Kamatani Y (2019). Comprehensive evaluation of structural variation detection algorithms for whole genome sequencing. Genome Biol.

[22] Genomics England Eligibility Criteria 2018.

[23] Landrum MJ, Lee JM, Benson M (2018). ClinVar: improving access to variant interpretations and supporting evidence. Nucleic Acids Res.

[24] Carss KJ, Arno G, Erwood M (2017). Comprehensive Rare Variant Analysis via Whole-Genome Sequencing to Determine the Molecular Pathology of Inherited Retinal Disease. Am J Hum Genet.

[25] https://www.biorxiv.org/content/10.1101/781088v1.

[26] Radziwon A, Arno G, Wheaton KD (2017). Single-base substitutions in the CHM promoter as a cause of choroideremia. Hum Mutat.

[27] Farazi Fard MA, Rebelo AP, Buglo E (2019). Truncating Mutations in UBAP1 Cause Hereditary Spastic Paraplegia. Am J Hum Genet.

[28] Wallmeier J, Frank D, Shoemark A (2019). De Novo Mutations in FOXJ1 Result in a Motile Ciliopathy with Hydrocephalus and Randomization of Left/Right Body Asymmetry. Am J Hum Genet.

[29] Cortese A (2020). Biallelic mutations in *SORD* cause a common and potentially treatable hereditary neuropathy with implications for diabetes. Nat Genet.

[30] Haendel MA, Chute CG, Robinson PN (2018). Classification, Ontology, and Precision Medicine. N Engl J Med.

[31] Eichler EE (2019). Genetic Variation, Comparative Genomics, and the Diagnosis of Disease. N Engl J Med.

[32] Yang Y, Muzny DM, Xia F (2014). Molecular findings among patients referred for clinical whole-exome sequencing. JAMA.

[33] Hu X, Li N, Xu Y (2018). Proband-only medical exome sequencing as a cost-effective first-tier genetic diagnostic test for patients without prior molecular tests and clinical diagnosis in a developing country: the China experience. Genet Med.

[34] Vissers LELM, van Nimwegen KJM, Schieving JH (2017). A clinical utility study of exome sequencing versus conventional genetic testing in pediatric neurology. Genet Med.

[35] Gilissen C, Hehir-Kwa JY, Thung DT (2014). Genome sequencing identifies major causes of severe intellectual disability. Nature.

[36] NHS national genomic test registry 2020.

