# Supplementary material for: The 100,000 Genomes Pilot on Rare Disease Diagnosis in Healthcare − A Preliminary Report
Source: N Engl J Med. Author manuscript; Available in PMC 2022 Aug 3. (PMC7613219; doi:10.1056/NEJMoa2035790)
Supplement: Supplement [file EMS151082-supplement-Supplement.pdf]

Supplementary Appendix

The 100,000 Genomes Project pilot: impact on rare disease diagnosis in a national healthcare system

Contents

Methods 3
Patient recruitment, sample collection and clinical data submission 3
DNA sequencing 4
Demographics 5
Diagnostic pipeline 5
Analysis of structural variants 7
Cohort-wide disease gene discovery 8
Healthcare benefits 9
Comparison to whole exome sequencing 9
Comparison to NHS Genomic Medicine Service Test Registry 10
Analysis of longitudinal, electronic healthcare records 10
References 13
Figure S1. Virtual panel-based variant filtering and prioritisation pipeline. 15
Figure S2: Totals of distinct activities during diagnostic odyssey and post genetic diagnosis periods. 16
Table S1. Clinical activity during diagnostic odyssey and post genetic diagnosis. 17
Table S2. Distribution of secondary care activity of unaffected relatives group: all activity, APC hospitalisations, outpatient appointments, accident and emergency visits, critical care attendances and diagnostic imaging events. 18
Table S3: Overview of 100,000 Genomes Project Pilot cohort costs of hospital care in the emergency department, inpatients, outpatients and critical care (intensive care) depicting costs for all participants, 2676 who are affected and 2146 unaffected people. 19
Table S4. Diagnostic yield by disease and family structure. 20
Table S5. Diagnostic yield by detailed prior genetic testing 28
Table S6. Diagnostic yield by disease category and most extensive type of prior genetic testing 29
Table S7. Diagnoses enabled by research analysis. 32
Table S8. Novel disease gene candidates. 36
Table S9. Reported healthcare benefits. 41
Table S10. Diagnostic odyssey for participant with a *TCN2* diagnosis. 45
Table S11: Diagnostic odyssey for participant with a *CTPS1* diagnosis. 46

Table S12. Pilot diagnostic yield for NHS Genomic Medicine Service clinical indications (>10 pilot cases available). 47

Methods

Patient recruitment, sample collection and clinical data submission

After ethical approval (National Research Ethics approvals 14/EE/1112 and 13/EE/032), rare disease participants were recruited and consented through 9 English NHS hospitals in partnership with the NIHR BioResource for Rare Diseases as part of the 100,000 Genomes Project Pilot. Eligible participants were nominated and identified by NHS consultants (recruiting clinicians), across a range of medical specialties from a broad spectrum of rare diseases. The selection of rare diseases was deliberately inclusive of disorders both a putative oligogenic basis and more complex aetiology as well as likely Mendelian single gene disorders to test the broad utility of whole genome sequencing. Additional family members, especially parents, were recruited where feasible according to the suggested guidelines for the specific disease. Recruitment was undertaken by a whole range of clinical practitioners (doctors, clinical/research nurses, geneticists, genetic counsellors, research practitioners) in the National Health Service and included patient media response and historical lists. Consent and collection of germline samples were secured using the preferred face to face route, during routine clinic appointments, inpatient admissions, or by a project specific appointment. In some circumstances a pre-arranged telephone call was used to obtained consent and postal bloods were dispatched. In all cases, the youngest affected member of the family with a sample collected was assigned as proband and family members were recruited following the proband. Where participants were under 16 years, recruitment took place in specialized children's facilities and parents legally consented along with assent from the child where possible. Those who were judged to have lost capacity were consented via advice from next of kin or with a professional consultee to ensure there was no loss of amenity to rare disease sufferers.

This pilot informed the 100,000 Genomes Project Main Programme where we have completed sequencing for 116,000 whole genomes including more than 83K from the rare disease patients and their families with the rest for cancer patients. These are still being validated for diagnostic reporting and we do not currently have the data for the comprehensive analysis presented here on the pilot for the Main Programme.

DNA sequencing

Samples were received as either DNA extracted from whole blood or as whole blood EDTA samples, which were used for extraction at the National Institutes for Health Research BioResource Laboratory in Cambridge. Samples were tested for adequate DNA concentration (Picogreen), quality controlled (QC) for degradation (gel electrophoresis) and purity (OD 260/280; Trinean) before selection for WGS. DNA samples were prepared at a minimum concentration of 30 ng/µl in 110 µl, visually inspected for degradation and had to have an OD 260/280 between 1.75 and 2.04. They were then prepared in batches of 96 and shipped on dry ice to the sequencing partner Illumina Laboratory Services (Illumina Inc, Great Chesterford, UK). Further sample QC was performed by Illumina Laboratory Services to ensure that the concentration of the DNA was > 30 ng/µl and that every sample generated high quality genotyping results (Illumina Infinium Human Core Exome microarray). Any samples with a repeated array genotyping call rate < 0.99, high levels of cross-contamination, mismatches with the declared gender that could not be resolved by further investigation, or for which consent had been withdrawn, were excluded from this study. The genotyping data were also used for positive sample identification and sample identity was verified before data delivery. In short, 0.5 µg of the DNA sample was fragmented using Covaris LE220 (Covaris Inc., Woburn, MA, USA) to obtain an average size of 450bp DNA fragments. DNA samples were processed using the Illumina TruSeq DNA Polymerase Chain Reaction-Free Sample Preparation kit (Illumina Inc., San Diego, CA, USA) on the Hamilton Microlab Star (Hamilton Robotics, Inc, Reno, NV, USA). The final libraries were checked using the Roche LightCycler 480 II (Roche Diagnostics Corporation, Indianapolis, IN, USA) with KAPA Library Quantification Kit (Kapa Biosystems Inc., Wilmington, MA, USA) for concentration. From February 2014 to June 2017, three read lengths were used: 100bp, 125bp and 150bp (377, 3,154 and 9,656 samples, respectively). Samples sequenced with 100bp and 125bp reads utilised three and two lanes of an Illumina HiSeq 2500 instrument, respectively, while samples sequenced with 150bp reads utilised a single lane of a HiSeq X instrument. At least 95% of the autosomal genome had to be covered at 15X and a maximum of 5% of insert sizes had to be less than twice the read length. Following sample and data QC at Illumina Laboratory Services, WGS data files were received at Genomics England for further QC. The WGS data returned by the sequencing provider underwent a series of processing steps. Briefly, contamination was estimated using VerifyBamID[1] and sex karyotypes were inferred from the ratios of mean X

and Y chromosome to automosomal coverage. Pairwise identity by descent coefficients were estimated within families using --genome from PLINK 1.9[2] and pairwise kinship coefficients across the cohort using the --kinship algorithm from KING[3]. This information was used to check for repeat sample submissions and sample swaps and consistency with reported family structure. A final dataset of 4,660 samples was obtained for downstream analysis.

Demographics

Demographic data for participants were collected from clinical notes or episodes, electronic patient records or from next of kin. To address incomplete record of self-reported ethnicity, we performed an analysis of the genomes to assign ancestry. Thirty thousand autosomal SNVs with a minor allele frequency > 5% and passing our site quality filters for depth, missingness, allelic imbalance and Hardy Weinberg Equilibrium deviations were selected based on presence in the participant genomes and the 1000 Genomes Project phase 3 where broad and fine-grained ancestry is known for each person. Principal components from the 1000 Genomes data were calculated and the 100KGP data projected onto these. A random forest model (randomForest R package, ntree=80, and a probability threshold of 90) based on 6 of the principal components and the known 5 super-populations described for the 1000 Genomes data (European, African, East Asian, South Asian and American) was trained and used to infer ancestry of our genomes (assigning the super-population with the highest probability from the model). The assigned ancestries showed a good correspondence with the available self-reported ethnicities for 435 probands where only 6 had conflicts.

Diagnostic pipeline

We created an automated pipeline to prioritise likely candidate variants based on virtual gene panels and this was used as the primary means of identifying diagnoses (Figure S1). This pipeline is described in more detail at https://www.genomicsengland.co.uk/?wpdmdl=15664 . Briefly, the pipeline first removes variants that do not segregate as expected for a causative variant in the family (or without strong evidence for being *de novo*), do not affect protein coding sequences, and those that are common (recessive disease models: > 1% in external and local datasets; dominant disease models: > 0.1% in ExAC[4], ESP_6500[5], UK10K[6] and local datasests or > 0.2% in GONL[7] and 1000g[8] datasets). Modes of segregation considered included

autosomal biallelic (simple recessive, compound heterozygous, uniparental isodisomy) and monoallelic (inherited or de novo), X-linked biallelic or monoallelic, and mitochondrial genome. Paternal and maternal imprinting were considered for autosomal monoallelic inherited mode for a small number of genes curated as relevant in PanelApp. *De novo* variant calling was performed using the relevant Platypus script with hard filters applied (maximum fraction of reads supporting the alternate allele in parent is 3%; minimum fraction of reads supporting the alternate allele in child is 20%; >=2 reads support the alternate allele in the child; results of a Bayesian factor method shows the likelihood that the variant is de novo is greater than the likelihood it is not de novo based on GL values for the trio and prior probability).

Variants affecting genes that are known to be involved in the proband's condition(s), including variants with the expected mode of inheritance for the gene and disease, are then prioritized by the application of virtual gene panels to the filtered results. These gene panels have been created for each recruited disease category through an expert, crowd-sourced review and internal curation process, facilitated by our PanelApp software.[9] Medical review of each family's pedigree and phenotypes was performed by our clinical team to assess whether additional panels beyond the recruited disease should be applied. They also assessed whether incomplete penetrance was a possibility or whether multiple monogenic conditions could be present. High impact variants or moderate impact *de novo* variants affecting genes in the applied panels were classified as tier 1 candidates, moderate impact variant types such as missense variants affecting the panel genes were otherwise classified as tier 2, and all other high or moderate impact filtered variants classified as tier 3. Identifying the appropriate panels for each case and keeping panels up to date is challenging, and in the case of larger panels such as for intellectual disability, can still result in many candidate variants.

To address these challenges and limit the possibility of overlooking, or inefficiently prioritising diagnoses, we also ran a parallel phenotype-based, variant prioritisation pipeline using the Exomiser software package (version 12.1.0 with 1909 databases).[10] Panel-based and Exomiser candidates were presented back to the recruiting clinicians in each regional Genomic Medicine Centre (GMC) via decision support systems provided by Congenica and Fabric Genomics. The latter also includes VAAST and Phevor, two gene prioritization algorithms

describing a gene burden test and using a proband's phenotype in genome analysis.[11,12] For cases processed by Congenica and Fabric Genomics, their clinical genetics teams also performed a review of each case and highlighted potentially pathogenic variants outside the gene panels that had been assigned at the medical review stage. Variants were reviewed by a clinical laboratory scientist in conjunction with the recruiting clinician and/or a clinical geneticist and classified according to the American College of Medical Genetics Guidelines.[13] Following variant review and classification, a diagnostic report was issued for each family. The final outcomes were captured in terms of whether a genetic diagnosis was obtained, the variants(s) involved and whether they explained all or just some of the phenotypes.

100KGP participants can opt in to receive secondary findings on predisposition to a range of cancers and familial hyperchosterolaemia but as we have not yet returned these yet at the request of the NHS we cannot report those results here.

Analysis of structural variants

MANTA[14] was used to call copy number (CNV) and structural variant (SV) calls, and CANVAS[15] used to identify >10 kB CNVs from our whole genome sequencing. Review of these calls was performed for the pilot cases, alongside analysis to either (i) look for a second CNV/SV variant in cases where a strong SNV candidate was identified in a bi-allelic gene on the applied panels, or (ii) systematically triage high quality CNV/SVs for all cases. For the latter, the calls first underwent quality control. Autosomal CANVAS calls were selected that passed the Illumina filters as well as hard filters on genotype quality, paired-end reads, repeat content, overlap problematic region, distance to the nearest segmental duplication, and distance to the nearest assembly gap. We used a novel random forest method for filtering of structural variants by quality of the calls. The training set consisted of 3,127,014 SV calls from 100 trios. For the purpose of training, a "high quality" SV call was defined as inherited (i.e. present in at least one parent) call with a population frequency below 10%. 80% of data was used for training and 20% for testing. The following variables were used to train the model and make the predictions: genotype quality, paired-end read support on left breakpoint, paired-end read support on right breakpoint, the genotype ('0/1' or '1/1'), FILTER value, presence of any overlap with Canvas call, the minimum repeat content between the two breakpoints, the maximum repeat content between the two breakpoints, the proportion of the call overlapping a "problematic" region, the distance to the nearest segmental duplication and

the distance to the nearest assembly gap (centromere/telomere and other gaps). Repeat content was taken from the Repeat Masker UCSC track. A "problematic" region was the region where >10% of the SV calls in the probands in the population were not present in at least one parent. The CANVAS and MANTA calls were further filtered to only those overlapping the exons and untranslated regions (UTRs) of genes in the applied panels, and internal frequency < 0.01. For monallelic genes, only MANTA heterozygous or CANVAS copy number = 1 or 3 calls were considered. For biallelic genes, only homozygous calls or cases where a second variant was observed in the other allele were kept. Finally, calls were visualized and those that appeared genuine were reviewed in multi-disciplinary team (MDT) sessions and validated by Sanger sequencing for smaller CNVs and array comparative genomic hybridization (CGH) for larger CNVs.

Cohort-wide disease gene discovery

A statistical framework was developed to detect enrichment of rare, predicted pathogenic variants in novel genes for specific diseases. The framework was run on all rare (< 0.1% dominant, < 1% recessive), coding variants that segregated with disease as expected for each possible mode of inheritance as filtered by Exomiser. A cohort was defined as all cases recruited under one of our specific disease categories and controls as all recruited probands except those under the broader category containing this disease in our rare disease hierarchy (Supplementary table 1) e.g. intellectual disability cases were compared to all non-neurological cases as controls.

To increase the power of this analysis we incorporated results from all 27,591 rare disease cases (57,002 genomes) available at the time of analysis (March 2019) in the 100KGP dataset. To maintain statistical validity and power, the analysis was limited to those disease-gene associations where at least five cases exist for the disease and where relevant variants in the gene were seen in at least four cases over the entire cohort. We used right-tailed Fisher's exact tests to assess the enrichment of variants under four separate scenarios: (i) rare, predicted damaging variants (Exomiser variant score > 0.8 corresponding to rare variants that are either loss of function (LoF) or missense variants predicted to be pathogenic by REVEL and/or MVP (ii) rare, predicted pathogenic variants, in a constrained coding region (CCR)[16], (iii) rare, predicted LoF variants, (iv) rare, *de novo* variants. For the latter, only trios or larger family structures where *de novo* calling was possible were considered. The Benjamini and

Hochberg method[17] was used to correct for multiple testing (total number of tests: 590,451); an overall false discovery rate adjusted (q-value) threshold of 0.10 was used for claiming significant gene-disease associations. In order to obtain a distribution of the number of discoveries under the null, we randomly permutated the case/control label 10,000 times and calculated the number of discoveries on the 10,000 permutated datasets using the same burden testing approach. Novel associations were defined as those involving genes that are not present in the PanelApp gene panel for the specific disease category (any level of evidence) and not in OMIM[18], for any related disorder. For associations being pursued further, such as the 22 associations described in Table S5, we searched PubMed to establish that there were not recent publications describing the associations.

Healthcare benefits

For a case to be closed and reported upon in the the 100KGP portal, an online form with built in validation has to be completed (reporting questionnaire) and data is therefore available for all cases. Details of the gene and variants involved in any diagnosis are collected alongside the resulting healthcare benefits in terms of any change in medication, additional surveillance for proband or relatives, clinical trial eligibility or whether it informs future reproductive choices. This data was queried in our internal database for all diagnoses reported in this study.

Comparison to whole exome sequencing

To allow a comparison of how many of our SNV/indel diagnoses from a WGS pipeline would have been detected by whole exome sequencing (WES) we analysed how many of the coding and non-coding SNV/indel diagnosed lay outside the regions detectable by such an approach. Exome targets were defined as those regions with at least 10x median coverage in gnomAD[19], (https://storage.googleapis.com/gnomad-public/release/2.1/coverage/exomes/gnomad.exomes.coverage.summary.tsv.bgz) or from 1000 samples from the Deciphering Developmental Disorders (DDD)[20] project that were all sequenced using the single WES capture kit (Matthew Hurles and Eugene Gardner, personal communication) as 10x median coverage is predicted to give 95% sensitivity.[21]

Use of these data in developing the NHS Genomic Medicine Service Test Registry

A test registry of clinical indications, where whole genome sequencing is the preferred diagnostic test, has recently been established by the NHS for its new Genomics Medicine Service (https://www.england.nhs.uk/publication/national-genomic-test-directories/). The 100KGP clinical team identified the recruited disease categories mapping to each of these indications to allow an estimation of likely diagnostic yield from WGS based on this study as shown in Table S8. This information informed selection of disorders for the NHS Genomic Test Directory.

Analysis of longitudinal, electronic healthcare records

The 100KGP consent model enables the collection of longitudinal healthcare records from registries and data repositories serviced nationally by NHS Digital and Public Health England. The primary function of these datasets is to provide epidemiological datasets, monitoring activity resource allocated to NHS clinical pathways to support commissioning of secondary care services (treatment provided by healthcare professionals who do not have the first contact with the patient). Standardized coding of diagnoses, procedures and interventions alongside quality assured demographic and administrative meta-data are also of high value to clinical research and medical decision support. Hospital Episode Statistics (HES), Diagnostic Imaging Dataset (DID) and Office of National Statistics (ONS) Mortality Register records were received by NHS Digital on a quarterly basis and covering the entirety of the 100KGP patients registered in England. The DID contains metadata collected from local radiology information systems (RIS) for a range of imaging activities from the year 2000 onwards. ONS mortality data with information - including cause of death – was extracted from the death certificate for all deaths registered in England and Wales since 1995. Our volume of HES publications includes:

- Admitted Patient Care (APC) records covering admitted patient care activity in English NHS hospitals and English NHS-commissioned activity in the independent sector from 1995 onwards.
- Outpatients (OP) records covering appointments at outpatient services from 2003 onwards.
- Adult Critical Care (ACC) records linking hospital admissions to use of critical care facilities from 2008 onwards.

- Accident and Emergency (AE) records covering attendance at Accident & Emergency units from 2007 onwards.

These data were then used to study the healthcare benefits achieved by genetic testing, and WGS in particular, by compiling the longitudinal activity for our cohort of rare disease patients to:

- Provide an overview of the probands' diagnostic odyssey.
- Assess impact on secondary healthcare resources, particularly in contrast to unaffected siblings.
- Highlight potential patterns in NHS hospital activity within disease categories.

A similar compendium of longitudinal activity for a control group of unaffected relatives from the UK 100,000 Genomes Project cohort was also compiled and used as a comparison. Due to the different commencement dates for collection of these datasets, longitudinal record analysis was restricted to probands with life-long activity captured in the datasets included. The cut-off birth year for the eligible patients was set to 2003 resulting in a total of 379 patients for the proband group and 318 for the young unaffected relatives group.

For each one of the participants in the longitudinal study the total number of records within APC, OP, AE, CC, DID datasets were assembled and ordered chronologically. Presence or absence of an entry in the mortality outcomes (ONS) dataset was checked. The assembled activity was inspected by a team of clinicians to mark the record signifying the likely start of odyssey (LSO) of the proband's engagement with healthcare services. The second milestone highlighted for the patient's journey was the date when genetic diagnosis (GD) was achieved, or for the unresolved cases, the last date of HES data we had available (31/07/2019). The same date (31/07/2019) was also the overall cut-off date for the longitudinal study. Using these time points, three periods of interest were defined for the 172 out of 379 patients in the proband group that had received a diagnosis, in line with the study's objectives:

1. Birth to likely start of odyssey (LSO).
2. LSO to Resolution/Genetic Diagnosis (or cut-off date)
3. Resolution to cut-off date.

For the control group of unaffected relatives only the period of clinical activity from birth to cut-off date was defined.

Clinical activity was grouped by type - hospital admissions (APC), outpatient appointment (OP), attendance to A&E department (AE), episodes in a critical care unit (CC), order of diagnostic image (DID) - every record counting as one episode. The medians were extracted for each of the groups, as well as a block for each period of interest. For the proband group, summaries are available for the whole group as well as for diseases that number more than 5 probands, both split by whether GMC decision (following genetic diagnosis) was achieved or not by the time of publication.

In addition to the longitudinal analysis of electronic health records, the cost of all secondary care for the cohorts of probands and unaffected relatives was calculated. This analysis was based on the available HES data (admitted patient care, outpatient attendances, accident and emergency presentations and critical care admissions), not the data from the DID. Each of the HES datasets was cleaned to facilitate processing through the Healthcare Resource Group (HRG) Reference Costs Grouper.[22] This software uses an algorithm that creates a hierarchy of mandatory/required fields from each HES dataset to group patient activity and derive appropriate HRG codes for each observation. Following the assignment of HRG codes to each observation, unit costs from the National Schedule of Reference Costs[23] were also attached. As the study period encompassed data over multiple years, we used the same HRG grouping algorithm for data in all years to ensure that changes in costs were from changes in utilisation and not from changes within the HRG algorithm or reference costs. We therefore used the HRG4+ 2016/17 Reference Costs Grouper and the 2016/17 National Schedule of Reference Costs. We then summarised the secondary care costs for the two cohorts both overall, and by type of care, calculating mean and median values.

References

1. Jun G, Flickinger M, Hetrick KN, et al. Detecting and estimating contamination of human DNA samples in sequencing and array-based genotype data. *Am J Hum Genet*. 2012;91(5):839-848.
2. Chang CC, Chow CC, Tellier LC, Vattikuti S, Purcell SM, Lee JJ. Second-generation PLINK: rising to the challenge of larger and richer datasets. *Gigascience*. 2015;4:7.
3. Manichaikul A, Mychaleckyj JC, Rich SS, Daly K, Sale M, Chen WM. Robust relationship inference in genome-wide association studies. *Bioinformatics*. 2010;26(22):2867-2873.
4. Karczewski KJ, Weisburd B, Thomas B, et al. The ExAC browser: displaying reference data information from over 60 000 exomes. Nucleic Acids Res. 2017;45(D1):D840-D845.
5. Exome Variant Server, NHLBI GO Exome Sequencing Project (ESP), Seattle, WA (URL: http://evs.gs.washington.edu/EVS/)
6. UK10K project. https://www.uk10k.org/
7. Boomsma DI, Wijmenga C, Slagboom EP, et al. The Genome of the Netherlands: design, and project goals. Eur J Hum Genet. 2014;22(2):221-227.
8. A global reference for human genetic variation, The 1000 Genomes Project Consortium, Nature 526, 68-74 (01 October 2015) doi:10.1038/nature15393.
9. Martin, A.R., Williams, E., Foulger, R.E. et al. PanelApp crowdsources expert knowledge to establish consensus diagnostic gene panels. Nat Genet **51,** 1560–1565 (2019).
10. Smedley D, Jacobsen JO, Jäger M, et al. Next-generation diagnostics and disease-gene discovery with the Exomiser. Nat Protoc. 2015 Dec;10(12):2004-15.
11. Yandell M, Huff C, Hu H, et al. A probabilistic disease-gene finder for personal genomes. Genome Res. 2011;21(9):1529-1542.
12. Singleton MV, Guthery SL, Voelkerding KV, et al. Phevor combines multiple biomedical ontologies for accurate identification of disease-causing alleles in single individuals and small nuclear families. Am J Hum Genet. 2014;94(4):599-610.
13. Richards S, Aziz N, Bale S, Bick D et al; ACMG Laboratory Quality Assurance Committee. Standards and guidelines for the interpretation of sequence variants: a joint consensus recommendation of the American College of Medical Genetics and Genomics and the Association for Molecular Pathology. Genet Med. 2015; 17(5):405-24. doi: 10.1038/gim.2015.30.
14. Zhang L, Bai W, Yuan N, Du Z. Comprehensively benchmarking applications for detecting copy number variation. PLoS Comput Biol. 2019 May 28;15(5):e1007069. doi: 10.1371/journal.pcbi.1007069. eCollection 2019 May. Erratum in: PLoS Comput Biol. 2019 Sep 20;15(9):e1007367.
15. Kosugi S, Momozawa Y, Liu X, Terao C, Kubo M, Kamatani Y. Comprehensive evaluation of structural variation detection algorithms for whole genome sequencing. Genome Biol. 2019 Jun 3;20(1):117.
16. Havrilla JM, Pedersen BS, Layer RM, Quinlan AR. A map of constrained coding regions in the human genome. Nat Genet. 2019 Jan;51(1):88-95.
17. Benjamini Y, Hochberg Y. Controlling the false discovery rate: a practical and powerful approach to multiple testing. Journal of the Royal Statistical Society, Series B. 1995. 57 (1): 289–300.
18. Online Mendelian Inheritance in Man, OMIM®. McKusick-Nathans Institute of Genetic Medicine, Johns Hopkins University (Baltimore, MD). https://omim.org/

19. Karczewski, K.J., Francioli, L.C., Tiao, G. *et al.* The mutational constraint spectrum quantified from variation in 141,456 humans. Nature 581**,** 434–443 (2020).
20. Wright CF, Fitzgerald TW, Jones WD, et al. Genetic diagnosis of developmental disorders in the DDD study: a scalable analysis of genome-wide research data. Lancet. 2015;385(9975):1305-1314.
21. Meynert AM, Bicknell LS, Hurles ME, Jackson AP, Taylor MS. Quantifying single nucleotide variant detection sensitivity in exome sequencing. BMC Bioinformatics.2013 Jun 18;14:195.
22. National Casemix Office. *Grouper User Manual - HRG4+ 2016/17 Reference Costs Grouper*. Health and Social Care Information Centre (hscic): England, 2017.
23. NHS Improvement. Reference costs 2016/17: highlights, analysis and intorduction to the data. NHS Improvement: London, 2017.
24. StringDB database: https://string-db.org/ accessed December 15th, 2019.
25. International Mouse Phenotyping Consortium portal: https://www.mousephenotype.org/ accessed August 30th, 2019.
26. Mouse Genome Database: http://www.informatics.jax.org/ accessed August 30th, 2019.

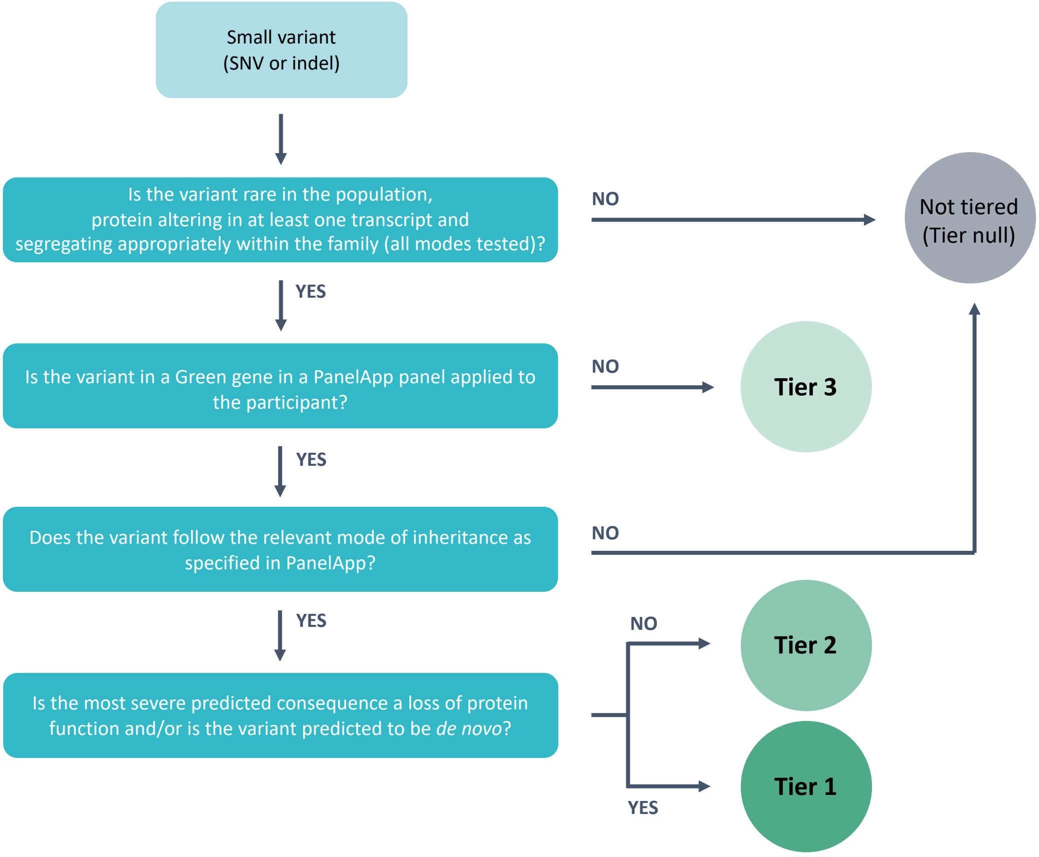

Figure S1. Virtual panel-based variant filtering and prioritisation pipeline.

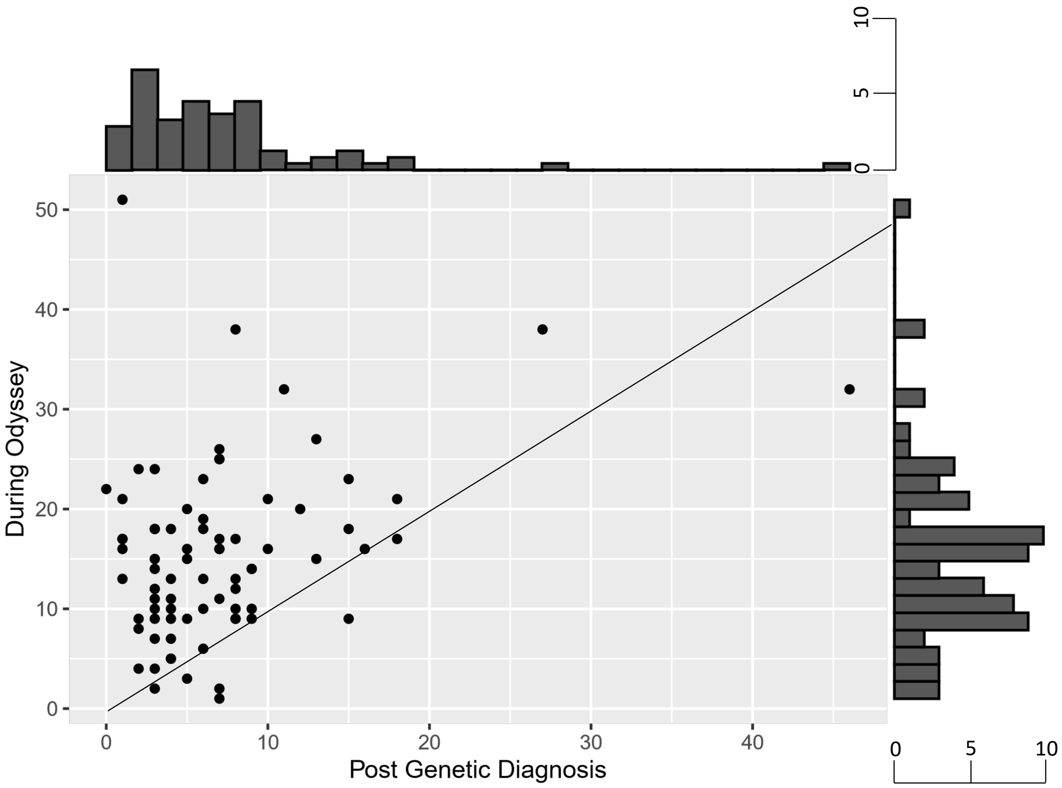

Figure S2: Totals of distinct activities during diagnostic odyssey and post genetic diagnosis periods.

Each dot represents a patient in the group and the coordinates of the dot correspond to the totals of their distinct clinical activity (main specialty of consultant seen in outpatients or during hospital admission and diagnostic imaging modality) during the diagnostic odyssey and the period after the genetic diagnosis. The marginal distributions on the right and above the plot show the resulting distribution of distinct activities for all the young probands who received a genetic diagnosis for the period following this milestone, and the period preceding it, respectively. The clinical activities captured in a electronic health record are often of the same type and may be repeated over time. Less variability in the type may indicate that the patient is settled in a healthcare pathway as a result of a conclusive diagnosis.

Table S1. Clinical activity during diagnostic odyssey and post genetic diagnosis.

Medians, dispersion (IQR) and monthly averages are shown for total clinical activity as well as subdivided by Admitted Patient Care (APC), Outpatient (OP) and Diagnostic Imaging Dataset (DID). Data was collected on 172 diagnosed probands who were born during or after 2006 for a median period of 75 months during their diagnostic odyssey and a median period of 18 months post diagnosis.

	During Diagnostic Odyssey			Post Genetic Diagnosis Established		
	**Median**	**IQR**	**Median Per Month**	**Median**	**IQR**	**Median Per Month**
**Total**	67.50	77.5	0.99	17.75	14.0	0.89
**APC**	11.50	6.0	0.08	1.25	0.0	0.00
**OP**	59.75	61.0	0.73	13.00	9.5	0.60
**DID**	7.25	5.0	0.06	2.00	0.0	0.00

Table S2. Distribution of secondary care activity of unaffected relatives group born during or after 2006: all activity, APC hospitalisations, outpatient appointments, accident and emergency visits, critical care attendances and diagnostic imaging events.

Data was collected on 318 unaffected relatives from birth to the study cut-off date with a median period of 10 years (120 months).

	IQR	Median
**Total**	32.00	18.0
**APC**	4.00	3.0
**OP**	22.00	11.0
**AE**	6.00	4.0
**CC**	1.25	2.5
**DID**	5.00	3.0

Table S3: Overview of 100,000 Genomes Project Pilot cohort costs of hospital care in the emergency department, inpatients, outpatients and critical care (intensive care) depicting costs for all participants, 2676 affected and 2146 unaffected people from 2183 familes.

Statistic	Category of care				
	Critical care	Emergency care [1]	Inpatient care [2]	Outpatient care	All care
**Number of episodes**					
All participants	347	16,696	43,714	177,125	**237,882**
Affected	303	11,875	34,143	136,952	**183,273**
Unaffected	44	4,765	9,446	39,451	**53,706**
Not known	0	56	125	722	**903**
**Total cost**					
All participants	£2,050,583	£2,530,399	£77,276,982	£26,243,354	**£108,101,318**
Affected	£1,811,555	£1,823,143	£62,525,278	£20,891,363	**£87,051,339**
Unaffected	£239,028	£700,259	£14,554,200	£5,254,317	**£20,747,803**
Not known	-	£6,997	£197,504	£97,674	**£302,176**
**Mean (median) cost per participant**					
All participants	£420 (£0)	£519 (£211)	£15,852 (£5,258)	£5,383 (£2,887)	**£22,175 (£9,231)**
Affected	£677 (£0)	£681 (£341)	£23.365 (£8,489)	£7,807 (£5,175)	**£32,530 (£15,310)**
Unaffected	£111 (£0)	£326 (£119)	£6,782 (£2,444)	£2,448 (£1,067)	**£9,668 (£4,285)**
Not known	£0 (£0)	£137 (£0)	£3,873 (£0)	£1,915 (£584)	**£5,925 (£1,078)**
Mean difference, affected versus unaffected (95% CI)	*£566 (£260 to £871)*	*£355 (£294 to £416)*	*£16,583 (£13,526 to £19,641)*	*£5,359 (£4,866 to £5,851)*	***£22,862 (£19,457 to £26,268)***

Table S4. Diagnostic yield by disease and family structure.

Disease area	Disease	Likely monogenic	Closed cases	Diagnostic yield %	Singletons	Diagnostic yield % (singletons)	Duos	Diagnostic yield % (duos)	Trios	Diagnostic yield % (trios)	Quads	Diagnostic yield % (quads)	Larger fams	Diagnostic yield % (larger fams)
Tumour syndromes	Multiple bowel polyps	N	134	3.0	123	2.4	4	0	7	14.3	0	NA	0	NA
Neurology and neurodevelopmental	Intellectual disability	Y	132	40.1	12	25	24	41.7	82	45.1	11	27.3	3	33.3
Ophthalmological	Rod-cone dystrophy	Y	115	47.8	23	34.8	18	50	67	55.2	6	16.7	1	0
Neurology and neurodevelopmental	Hereditary ataxia	Y	120	28.5	62	21.0	19	36.9	34	32.4	4	50	1	0
Neurology and neurodevelopmental	Charcot-Marie-Tooth disease	Y	77	35.1	42	33.3	14	42.8	18	33.3	3	33.3	0	NA
Metabolic	Mitochondrial disorders	Y	76	40.2	23	21.7	7	28.6	39	50..4	6	50	1	100
Neurology and neurodevelopmental	Hereditary spastic paraplegia	Y	74	33.8	34	20.6	11	63.6	24	33.3	1	100	4	50
Hearing and ear	Congenital hearing impairment (profound/severe)	Y	54	46.3	12	16.7	8	62.5	28	59.3	5	40	1	0
Renal and urinary tract	Renal tract calcification (or Nephrolithiasis/nephrocalc inosis)	N	46	4.3	37	5.4	4	0	4	0	0	NA	1	0
Endocrine	Kallmann syndrome	N	43	20.9	36	19.4	6	33.3	1	0	0	NA	0	NA
Renal and urinary tract	CAKUT	N	43	7.0	27	7.4	6	0	7	14.3	2	0	0	NA
Tumour syndromes	Familial colon cancer	N	37	5.4	33	6.1	2	0	2	0	0	NA	0	NA
Tumour syndromes	Multiple endocrine tumours	N	36	2.8	29	3.5	4	0	3	0	0	NA	0	NA
Ciliopathies	Non-CF bronchiectasis	N	38	5.3	23	4.4	7	14.3	7	0	1	0	0	NA
Ophthalmological	Inherited optic neuropathies	Y	33	33.3	19	36.8	8	37.5	6	16.7	0	NA	0	NA
Ophthalmological	Cone Dysfunction Syndrome	Y	33	39.8	5	40	8	25	22	45.4	0	NA	0	NA
Cardiovascular	Familial Thoracic Aortic Aneurysm Disease	N	29	10.3	13	15.4	9	11.1	4	0	2	0	1	0

Renal and urinary tract	Cystic kidney disease	Y	27	22.2	23	26.1	2	0	2	0	0	NA	0	NA
Rheumatological	Ehlers-Danlos syndrome type 3	N	26	11.5	11	0	4	0	7	28.6	3	33.3	1	0
Tumour syndromes	Familial breast cancer	N	26	3.8	21	4.8	4	0	1	0	0	NA	0	NA
Tumour syndromes	Neuro-endocrine Tumours- PCC and PGL	N	29	6.9	16	12.5	9	0	4	0	0	NA	0	NA
Dermatological	Severe multi-system atopic disease with high IgE	N	24	4.2	5	0	4	0	14	7.1	1	0	0	NA
Neurology and neurodevelopmental	Congenital myopathy	Y	25	16.7	9	0	4	25	9	33.3	2	0	1	0
Ophthalmological	Posterior segment abnormalities	Y	29	55.2	5	40	9	33.3	11	81.2	3	33.3	1	100
Cardiovascular	Dilated Cardiomyopathy (DCM)	N	25	24.0	12	16.7	7	42.3	9	11.1	1	0	0	NA
Ophthalmological	Cataracts	Y	23	47.8	13	69.2	3	0	6	33.3	1	0	0	NA
Neurology and neurodevelopmental	Early onset dystonia	Y	30	23.3	6	0	4	25	17	17.6	1	100	0	NA
Renal and urinary tract	Unexplained kidney failure in young people	N	20	30	16	12.5	2	100	2	100	0	NA	0	NA
Ophthalmological	Inherited macular dystrophy	Y	19	47.4	3	0	2	100	13	53.8	1	0	0	NA
Metabolic	Ketotic hypoglycaemia	N	18	16.7	1	0	4	25	12	16.7	1	0	0	NA
Neurology and neurodevelopmental	Paediatric motor neuronopathies	Y	24	33.3	3	0	4	50	15	26.7	2	100	0	NA
Ophthalmological	Leber Congenital Amaurosis / Early-Onset Severe Retinal Dystrophy	Y	16	56.2	1	100	0	NA	15	53.3	0	NA	0	NA
Skeletal	Osteogenesis Imperfecta	Y	20	45	4	50	6	33.3	5	40	4	75	1	0
Rheumatological	Juvenile dermatomyositis	N	15	6.7	1	100	2	0	12	0	0	NA	0	NA
Skeletal	Unexplained skeletal dysplasia	Y	14	7.1	2	0	1	0	8	12.5	3	0	0	NA
Cardiovascular	Hypertrophic Cardiomyopathy	N	18	44.4	12	25	1	0	5	100	0	NA	0	NA
Gastroenterological	Infantile enterocolitis & monogenic inflammatory bowel disease	N	21	4.8	0	NA	1	0	14	7.1	5	0	1	0
Dysmorphic and congenital abnormality syndromes	VACTERL-like phenotypes	N	14	0	10	0	2	0	2	0	0	NA	0	NA
Ciliopathies	Bardet-Biedl Syndrome	Y	14	57.1	1	0	6	66.7	6	66.7	1	0	0	NA

Neurology and neurodevelopmental	Distal myopathies	Y	17	17.7	13	15.4	1	0	2	50	0	NA	1	0
Ciliopathies	Primary ciliary disorders	Y	13	23.1	10	20	3	33.3	0	NA	0	NA	0	NA
Skeletal	Stickler syndrome	Y	13	30.8	8	12.5	1	0	3	100	1	0	0	NA
Neurology and neurodevelopmental	Epileptic encephalopathy	Y	16	43.8	0	NA	3	100	11	36.3	2	0	0	NA
Neurology and neurodevelopmental	Classical tuberous sclerosis	Y	11	9.1	9	0	0	NA	2	50	0	NA	0	NA
Endocrine	IUGR and IGF abnormalities	Y	11	0	1	0	3	0	5	0	2	0	0	NA
Neurology and neurodevelopmental	Complex Parkinsonism (includes pallido-pyramidal syndromes)	N	12	16.7	8	12.5	2	0	2	50	0	NA	0	NA
Renal and urinary tract	Proteinuric renal disease	N	11	45.5	7	42.9	1	100	3	33.3	0	NA	0	NA
Cardiovascular	Brugada syndrome	N	17	5.9	7	0	3	0	4	25	1	0	0	NA
Renal and urinary tract	Familial haematuria	N	11	18.1	8	0	1	100	2	50	0	NA	0	NA
Cardiovascular	Long QT syndrome	N	13	23.1	7	28.6	1	0	4	0	0	NA	1	100
Neurology and neurodevelopmental	Skeletal Muscle Channelopathies	N	13	7.7	6	16.7	2	0	5	0	0	NA	0	NA
Neurology and neurodevelopmental	Congenital muscular dystrophy	Y	12	33.3	5	60	1	0	5	0	1	100	0	NA
Endocrine	Familial or syndromic hypoparathyroidism	Y	9	44.4	6	50	2	50	1	0	0	NA	0	NA
	Ocular and oculo-cutaneous albinism	Y	9	55.6	1	100	1	0	7	57.1	0	NA	0	NA
Gastroenterological	Infantile pseudo-obstruction	N	9	0	0	NA	0	NA	6	0	3	0	0	NA
Hearing and ear	Bilateral microtia	Y	9	22.2	1	0	1	100	5	20	1	0	1	0
Endocrine	Congenital adrenal hypoplasia	Y	10	33.3	3	33.3	2	100	3	0	1	0	1	0
Neurology and neurodevelopmental	Inherited white matter disorders	Y	11	18.2	2	0	1	0	6	17.6	2	50	0	NA
Neurology and neurodevelopmental	Limb girdle muscular dystrophy	Y	8	25	5	40	1	0	1	0	1	0	0	NA
Ophthalmological	Rod Dysfunction Syndrome	Y	8	50	2	50	1	100	4	25	1	100	0	NA
Neurology and neurodevelopmental	Brain channelopathy	N	8	12.5	4	0	0	NA	4	25	0	NA	0	NA
Ophthalmological	Corneal abnormalities	N	9	11.1	1	0	3	0	5	20	0	NA	0	NA

Neurology and neurodevelopmental	Early onset and familial Parkinson's Disease	N	10	20	3	0	2	50	5	20	0	NA	0	NA
Cardiovascular	Familial non-syndromic congenital heart disease	N	8	12.5	1	0	2	0	3	0	1	100	1	0
	Agranulocytosis	Y	7	14.3	3	0	2	50	2	0	0	NA	0	NA
Neurology and neurodevelopmental	Familial Genetic Generalised Epilepsies	N	7	0	3	0	2	0	1	0	1	0	0	NA
Neurology and neurodevelopmental	Kleine-Levin syndrome	N	7	0	0	NA	1	0	6	0	0	NA	0	NA
Renal and urinary tract	Renal tubular acidosis	N	7	14.3	3	33.3	1	0	2	0	1	0	0	NA
	A- or hypo-gammaglobulinaemia	Y	6	33.3	0	NA	2	0	3	33.3	0	NA	1	100
Skeletal	Craniosynostosis syndromes phenotypes	Y	7	28.6	1	0	1	100	5	20	0	NA	0	NA
Ophthalmological	Familial exudative retinopathy	Y	6	33.3	0	NA	2	0	4	50	0	NA	0	NA
Dysmorphic and congenital abnormality syndromes	RASopathies	Y	6	16.7	1	0	0	NA	4	0	1	100	0	NA
Cardiovascular	Arrhythmogenic Right Ventricular Cardiomyopathy	N	13	23.1	5	20	4	NA	3	33.3	1	100	0	NA
Tumour syndromes	Familial Tumours Syndromes of the central & peripheral Nervous system	N	6	66.7	4	75	2	50	0	NA	0	NA	0	NA
Tumour syndromes	Multiple Tumours	N	6	16.7	4	25	1	0	1	0	0	NA	0	NA
Ophthalmological	Anophthalmia/microphthamia	Y	5	40	0	NA	1	100	4	25	0	NA	0	NA
Ophthalmological	Ocular coloboma	Y	5	20	0	NA	0	NA	5	20	0	NA	0	NA
Ciliopathies	Rare multisystem ciliopathy disorders	Y	5	40	0	NA	1	100	3	0	1	100	0	NA
Neurology and neurodevelopmental	Amyotrophic lateral sclerosis/motor neuron disease	N	5	20	2	0	1	100	2	0	0	NA	0	NA
Neurology and neurodevelopmental	Early onset dementia (encompassing fronto-temporal dementia and prion disease)	N	5	20	5	20	0	NA	0	NA	0	NA	0	NA
Endocrine	Familial diabetes	N	5	0	5	0	0	NA	0	NA	0	NA	0	NA

Cardiovascular	Left Ventricular Noncompaction Cardiomyopathy	N	5	20	4	25	0	NA	1	0	0	NA	0	NA
Neurology and neurodevelopmental	Moyamoya disease	N	5	0	0	NA	0	NA	4	0	1	0	0	NA
Ophthalmological	Glaucoma (developmental)	Y	4	50	0	NA	1	100	3	33.3	0	NA	0	NA
Renal and urinary tract	Atypical haemolytic uraemic syndrome	N	4	25	0	NA	1	100	3	0	0	NA	0	NA
Cardiovascular	Catecholaminergic Polymorphic Ventricular Tachycardia	N	4	0	1	0	0	NA	3	0	0	NA	0	NA
Dermatological	Erythropoietic protoporphyria, mild variant	N	4	25	2	50	0	NA	2	0	0	NA	0	NA
Renal and urinary tract	Extreme early-onset hypertension	N	4	0	4	0	0	NA	0	NA	0	NA	0	NA
Tumour syndromes	Genodermatoses with malignancies	N	4	25	3	33.3	1	0	0	NA	0	NA	0	NA
Dermatological	Hydroa vacciniforme	N	4	0	1	0	0	NA	3	0	0	NA	0	NA
Neurology and neurodevelopmental	Arthrogryposis	Y	4	0	0	NA	2	0	1	NA	1	0	0	NA
Haematological	Congenital anaemias	Y	5	20	0	NA	1	0	2	0	2	50	0	NA
Ophthalmological	Developmental macular and foveal dystrophy	Y	3	0	0	NA	0	NA	3	0	0	NA	0	NA
Endocrine	Disorders of sex development	Y	3	66.7	2	50	0	NA	1	100	0	NA	0	NA
Metabolic	Mucopolysacharideosis, Gaucher, Fabry	Y	3	33.3	1	0	0	NA	1	100	1	0	0	NA
Metabolic	Undiagnosed metabolic disorders	Y	3	66.7	1	0	0	NA	1	100	0	NA	1	100
Neurology and neurodevelopmental	Cerebrovascular disorders	N	4	0	0	NA	2	0	1	0	1	0	0	NA
	Familial dysautonomia	N	3	0	0	NA	1	0	2	0	0	NA	0	NA
	Multiple lipomas	N	3	0	2	0	1	0	0	NA	0	NA	0	NA
	Regional overgrowth disorders	N	3	0	2	0	1	0	0	NA	0	NA	0	NA
Hearing and ear	Autosomal dominant deafness	Y	2	50	0	NA	1	0	1	100	0	NA	0	NA
Dermatological	Autosomal recessive congenital ichthyosis	Y	2	0	0	NA	0	NA	1	0	1	0	0	NA
Growth	Beckwith-Wiedemann syndrome (BWS) and other	Y	2	50	1	100	0	NA	1	0	0	NA	0	NA

	congenital overgrowth disorders													
Respiratory	Hereditary haemorrhagic telangiectasia	Y	2	0	1	0	0	NA	1	0	0	NA	0	NA
Ophthalmological	Infantile nystagmus	Y	2	50	0	NA	1	100	1	0	0	NA	0	NA
Neurology and neurodevelopmental	Rhabdomyolysis and metabolic muscle disorders	Y	2	0	0	NA	1	0	1	0	0	NA	0	NA
	SCID	Y	2	50	0	NA	0	NA	2	50	0	NA	0	NA
Skeletal	Thoracic dystrophies	Y	2	0	0	NA	0	NA	2	0	0	NA	0	NA
Endocrine	Congenital hypothyroidism or thyroid agenesis	N	2	0	2	0	0	NA	0	NA	0	NA	0	NA
Dermatological	Ectodermal dysplasia without a known gene mutation	N	2	50	0	NA	0	NA	2	50	0	NA	0	NA
Rheumatological	Ehlers-Danlos syndromes	N	6	16.7	2	50	2	0	2	0	0	NA	0	NA
Cardiovascular	Fallots tetralogy	N	2	0	0	NA	0	NA	2	0	0	NA	0	NA
Cardiovascular	Familial cerebral small vessel disease	N	2	50	2	50	0	NA	0	NA	0	NA	0	NA
Neurology and neurodevelopmental	Familial Focal Epilepsies	N	2	0	0	NA	1	0	1	0	0	NA	0	NA
Endocrine	Hyperinsulinism	N	2	50	0	NA	1	100	1	0	0	NA	0	NA
Haematological and immunological	Monogenic thrombophilia	N	2	0	1	0	1	0	0	NA	0	NA	0	NA
Tumour syndromes	Rare tumour predisposition syndromes	N	2	0	2	0	0	NA	0	NA	0	NA	0	NA
Cardiovascular	Dilated Cardiomyopathy and conduction defects		2	50	2	50	0	NA	0	NA	0	NA	0	NA
Neurology and neurodevelopmental	Cerebellar hypoplasia	Y	1	100	0	NA	0	NA	1	100	0	NA	0	NA
Skeletal	Choanal atresia	Y	1	0	0	NA	0	NA	0	NA	1	0	0	NA
Growth	Classical Beckwith-Wiedemann syndrome	Y	1	0	1	0	0	NA	0	NA	0	NA	0	NA
	Combined B and T cell defect	Y	1	0	0	NA	1	0	0	NA	0	NA	0	NA
Neurology and neurodevelopmental	Congenital myaesthenia	Y	1	0	0	NA	1	0	0	NA	0	NA	0	NA
Hearing and ear	Ear malformations	Y	1	0	0	NA	0	NA	1	0	0	NA	0	NA
Haematological	Early onset pancytopenia and red cell disorders	Y	1	0	0	NA	0	NA	1	0	0	NA	0	NA

Hearing and ear	Familial hemifacial microsomia	Y	1	0	0	NA	1	0	0	NA	0	NA	0	NA
Cardiovascular	Familial hypercholesterolaemia	Y	1	0	1	0	0	NA	0	NA	0	NA	0	NA
Neurology and neurodevelopmental	Holoprosencephaly	Y	1	0	1	0	0	NA	0	NA	0	NA	0	NA
Metabolic	Hyperammonaemia	Y	1	0	0	NA	0	NA	0	NA	1	0	0	NA
Haematological and immunological	Inherited bleeding disorders	Y	1	0	0	NA	1	0	0	NA	0	NA	0	NA
Neurology and neurodevelopmental	Intracerebral calcification disorders	Y	1	0	0	NA	1	0	0	NA	0	NA	0	NA
Rheumatological	Kyphoscoliotic Ehlers-Danlos syndrome	Y	1	0	0	NA	0	NA	1	0	0	NA	0	NA
Endocrine	Significant early-onset obesity +/- other endocrine features and short stature	Y	1	0	0	NA	0	NA	1	0	0	NA	0	NA
	Balanced translocations with an unusual phenotype	N	3	33.3	1	0	0	NA	2	50	0	NA	0	NA
Neurology and neurodevelopmental	Cerebral arteriovenous malformations	N	1	0	0	NA	1	0	0	NA	0	NA	0	NA
Neurology and neurodevelopmental	Cerebral vascular malformations	N	1	100	1	100	0	NA	0	NA	0	NA	0	NA
	Currarino triad	N	1	0	0	NA	1	0	0	NA	0	NA	0	NA
	Familial Neural Tube Defects	N	1	0	0	NA	0	NA	1	0	0	NA	0	NA
Tumour syndromes	Familial prostate cancer	N	1	100	1	100	0	NA	0	NA	0	NA	0	NA
Haematological and immunological	Inherited platelet disorders	N	1	0	0	NA	1	0	0	NA	0	NA	0	NA
Dermatological	Palmoplantar keratoderma and erythrokeratodermas	N	1	0	0	NA	1	0	0	NA	0	NA	0	NA
Neurology and neurodevelopmental	Parkinson Disease and Complex Parkinsonism	N	1	0	1	0	0	NA	0	NA	0	NA	0	NA
Rheumatological	Periodic fever syndromes	N	1	0	0	NA	0	NA	1	0	0	NA	0	NA
	Pityriasis rubra pilaris	N	1	0	1	0	0	NA	0	NA	0	NA	0	NA
Endocrine	Resistance to thyroid hormone	N	1	0	1	0	0	NA	0	NA	0	NA	0	NA
Skeletal	Multiple Epiphyseal Dysplasia		1	0	0	NA	1	0	0	NA	0	NA	0	NA
Neurology and neurodevelopmental	Parkinson Disease and Complex Parkinsonism		1	0	1	0	0	NA	0	NA	0	NA	0	NA

Rheumatological	Periodic fever syndromes	1	0	0	NA	0	NA	1	0	0	NA	0	NA
Endocrine	Resistance to thyroid hormone	1	0	1	0	0	NA	0	NA	0	NA	0	NA
Endocrine	Significant early-onset obesity +/- other endocrine features and short stature	1	0	0	NA	0	NA	1	0	0	NA	0	NA
Growth	Silver Russell syndrome	1	0	0	NA	0	NA	1	0	0	NA	0	NA

452 **Table S5. Percentage Diagnostic uplift by detailed prior genetic testing type.**

Testing	Number of probands	% Diagnostic uplift
Single gene(s)	393	29
Single gene(s) mtDNA	21	42.9
Single gene(s) STR	72	22.2
mtDNA genome	25	36
Karyotyping	29	44.8
Fragile X	27	51.9
DNA methylation	12	50
ArrayCGH	10	40
Arrays	61	42.6
NGS panels	193	30.1
WES	16	37.5

Table S6. Percentage Diagnostic uplift by disease category and most extensive type of prior genetic testing

	Number of probands	% Diagnostic uplift
**Cardiovascular disorders**	64	25
None	29	20.7
Panel	18	33.3
Targeted	17	23.5
**Ciliopathies**	14	42.9
Chromosomal	2	100
None	3	100
Panel	9	11.1
**Dermatological disorders**	10	10
None	7	0
Targeted	3	33.3
**Dysmorphic and congenital abnormality syndromes**	5	0
Chromosomal	1	0
Panel	2	0
Targeted	2	0
**Endocrine disorders**	35	28.6
Chromosomal	2	50
None	27	22.2
Targeted	6	50
**Gastroenterological disorders**	28	3.6
Exome	1	0
None	21	0
Panel	4	0
Targeted	2	50
**Growth disorders**	2	0

Targeted	2	0
**Haematological disorders**	1	0
Targeted	1	0
**Hearing and ear disorders**	48	52.1
Chromosomal	4	25
None	28	53.6
Panel	7	71.4
Targeted	9	44.4
**Metabolic disorders**	80	41.3
Chromosomal	6	100
Exome	3	66.7
None	23	26.1
Panel	16	37.5
Targeted	32	40.6
**Neurology and neurodevelopmental disorders**	416	29.6
Chromosomal	26	42.3
Exome	5	20
None	147	29.3
Panel	60	28.3
Targeted	178	28.7
**Ophthalmological disorders**	287	47
Chromosomal	4	50
Exome	5	40
None	161	53.4
Panel	60	38.3
Targeted	57	38.6
**Renal and urinary tract disorders**	45	8.9
Chromosomal	4	25

None	29	3.4
Panel	2	50
Targeted	10	10
**Respiratory disorders**	1	0
None	1	0
**Rheumatological disorders**	25	4
Chromosomal	2	0
None	22	4.5
Targeted	1	0
**Skeletal disorders**	21	14.3
Chromosomal	1	0
None	11	9.1
Panel	1	0
Targeted	8	25
**Tumour syndromes**	75	9.3
Chromosomal	1	0
Exome	1	0
None	14	14.3
Panel	12	0
Targeted	47	10.6
**Unclassified**	20	30
Exome	1	100
None	13	30.8
Panel	3	0
Targeted	3	33.3
**Total**	1177	31.5

Table S7. Diagnoses enabled by research analysis.

Each row represents an independent case.

Category	Recruited disease	Gene	Variant(s)
Coding SNV/indel	Hereditary ataxia	*EBF3*	de novo ENST00000355311.5:c.530C>T:p.(Pro177Leu) variant detected in constrained coding region
Mitochondrial genome	Ketotic hypoglycaemia	*MT-ATP6*	m.9176T>G with 100% mutational load
Mitochondrial genome	Hereditary spastic paraplegia	*MT-ATP6*	m.9176T>G with 100% mutational load
Mitochondrial genome	Intellectual disability	*MT-ATP6*	m.8993T>G with 82% mutational load
Mitochondrial genome	Mitochondrial disorders	*MT-ND3*	m.10191T>C with 85% mutational load
Non-coding SNV/indel	Cone Dysfunction Syndrome	*ABCA4*	Known pathogenic (Clinvar VCV000092870.4), intronic variant (NM_000350.3(ABCA4):c.5461-10T>C) in compound heterozygosity with predicted damaging missense variant
Non-coding SNV/indel	Cone Dysfunction Syndrome	*ABCA4*	Known pathogenic (Clinvar VCV000092870.4), intronic variant (NM_000350.3(ABCA4):c.5461-10T>C) in compound heterozygosity with predicted damaging missense variant
Non-coding SNV/indel	Posterior segment abnormalities	*ABCA4*	Known pathogenic (Clinvar VCV000092870.4), intronic variant (NM_000350.3(ABCA4):c.5461-10T>C) in compound heterozygosity with predicted damaging missense variant
Non-coding SNV/indel	Posterior segment abnormalities	*ABCA4*	Known pathogenic (Clinvar VCV000092870.4), intronic variant (NM_000350.3(ABCA4):c.5461-10T>C) in compound heterozygosity with predicted damaging missense variant
Non-coding SNV/indel	Posterior segment abnormalities	*ABCA4*	Known pathogenic (Clinvar VCV000092870.4), intronic variant (NM_000350.3(ABCA4):c.5461-10T>C) in compound heterozygosity with predicted damaging missense variant
Non-coding SNV/indel	Posterior segment abnormalities	*ABCA4*	Known pathogenic (Clinvar VCV000092870.4), intronic variant (NM_000350.3(ABCA4):c.5461-10T>C) in compound heterozygosity with predicted damaging missense variant
Non-coding SNV/indel	Cone Dysfunction Syndrome	*ABCA4*	Known pathogenic intronic variant (ABCA4:ENST00000370225: c.5196+1137G>A) confirmed to disrupt splicing by in vitro assays. In compound heterozygosity with predicted damaging missense variant
Non-coding SNV/indel	Congenital muscular dystrophy	*COL6A1*	Known pathogenic (Clinvar VCV000542998.2) intronic variant (NM_001848.2(COL6A1):c.930+189C>T) observed as de novo
Non-coding SNV/indel	Multiple endocrine tumours	*MEN1*	Known pathogenic (Clinvar VCV000200981.3) intronic variant (NM_000244.3(MEN1):c.799-9G>A)
Non-coding SNV/indel	Hereditary spastic paraplegia	*POLR3A*	Known pathogenic (VCV000445922.2) intronic variant (NM_007055.4(POLR3A):c.1909+22G>A) in compound heterozygosity with LoF variant
Non-coding SNV/indel	Hereditary ataxia	*POLR3A*	Known pathogenic (VCV000445922.2) intronic variant (NM_007055.4(POLR3A):c.1909+22G>A) in compound heterozygosity with LoF variant

Non-coding SNV/indel	Congenital hearing impairment (profound/severe) and Posterior segment abnormalities	*USH2A*	Known pathogenic (VCV000030722.3) intronic variant (NM_206933.3(USH2A):c.7595-2144A>G) in compound heterozygosity with LoF variant
Non-coding SNV/indel	Cataracts	*EPHA2*	Known pathogenic (RCV000644433.1) intronic variant (NM_004431.5(EPHA2):c.2826-9G>A) with incomplete penetrance in unaffected mother
Non-coding SNV/indel	Congenital hearing impairment (profound/severe) and posterior segment abnormalities	*GUCY2D*	Novel 5'-UTR variant (NM_000180.3:c.-148T>C) confirmed to affect expression by in vitro assays. In compound heterozygosity with predicted damaging variant
Non-coding SNV/indel	Leber Congenital Amaurosis / Early-Onset Severe Retinal Dystrophy	*GUCY2D*	Novel 5'-UTR variant (NM_000180.3:c.-148T>C) confirmed to affect expression by in vitro assays. In compound heterozygosity with predicted damaging variant
Non-coding SNV/indel	Congenital hearing impairment (profound/severe) and posterior segment abnormalities	*USH2A*	Novel intronic variant (USH2A:ENST00000307340:c.12066+4409C>G) confirmed to disrupt splicing by in vitro assays. In compound heterozygosity with predicted damaging variant
Non-coding SNV/indel	Rod-cone dystrophy	*CRB1*	Novel intronic variant (ENST00000367400:c.3879-1203C>G) confirmed to disrupt splicing by in vitro assays
Non-coding SNV/indel	Rod-cone dystrophy	*CRB1*	Novel intronic variant (CRB1:ENST00000367400:c.4006-10A>G) confirmed to disrupt splicing by in vitro assays. In compound heterozygosity with predicted damaging variant
Non-coding SNV/indel	Rod-cone dystrophy	*CHM*	Novel hemizygous promoter variant (CHM:ENST00000357749.2:c.-98:G:A) confirmed to reduce expression by luciferase assay.[26]
Non-coding SNV/indel	Rod-cone dystrophy	*CHM*	Novel hemizygous intronic variant (CHM:ENST00000357749.2:c.315–1536A>G) confirmed to disrupt splicing by in vitro assays.[24]
Non-coding SNV/indel	Inherited optic neuropathies	*OPA1*	Novel intronic variant (OPA1:NM_130837.2:c.610+360G>A) confirmed to disrupt splicing by in vitro assays.
Non-coding SNV/indel	Retinitis pigmentosa	*PRPF31*	Novel intronic variant (PRPF31:ENST00000321030:c.1374+569 C>G) confirmed to splice in a new exon by RT-PCR and sequencing of patient sample.
Structural variant	Bardet-Biedl Syndrome	*BBS1*	Heterozygous deletion of exons 10-11 in compound heterozygosity with predicted damaging SNV

Structural variant	Corneal abnormalities	*SLC4A11*	Heterozygous deletion of whole gene in compound heterozygosity with predicted damaging SNV
Structural variant	Posterior segment abnormalities	*USH2A*	Heterozygous duplication of exons 57-60 in compound heterozygosity with predicted damaging SNV
Structural variant	Rod-cone dystrophy	*RP2*	Hemizygous deletion of exon 3 in male proband and heterozygous in affected mother
Structural variant	Rod-cone dystrophy	*CNGB1*	Homozygous deletion of exons 25-27 in proband and affected sib
Structural variant	Hereditary ataxia	*ITPR1*	Heterozygous deletion of exons 1-51 in proband and affected sib and father
Structural variant	Hereditary ataxia	*RARS2*	Heterozygous deletion of exon 10 in compound heterozygosity with predicted damaging SNV in proband and affected sib
Structural variant	Multiple Tumours	*MSH2*	Heterozygous duplication of exons 1-7 in tandem in compound heterozygosity with predicted damaging SNV
Structural variant	Mitochondrial disorders	*CEP78*	Heterozygous deletion of exons 1 to 5 in compound heterozygosity with predicted damaging SNV in proband and affected sib
Structural variant	Glaucoma (developmental)	*PBX1*	Heterozygous deletion of exons 3-4
Structural variant	Cystic kidney disease	*NPHP1*	Homozygous deletion of whole gene
Structural variant	Complex Parkinsonism (includes pallido-pyramidal syndromes)	*VPS13A*	Heterozygous deletion of exon 14 in compound heterozygosity with predicted damaging SNV
Structural variant	Kleine-Levin syndrome	*LPIN1*	Heterozygous deletion of exon 19 in compound heterozygosity with predicted damaging SNV
Structural variant	Hereditary ataxia	*ANO10*	Heterozygous deletion of exon 12 in compound heterozygosity with predicted damaging SNV
Structural variant	Atypical haemolytic uraemic syndrome	*CFH*	Heterozygous deletion of exons 20-22
Structural variant	Hereditary ataxia	*ITPR1*	Heterozygous deletion of whole gene
Structural variant	Cystic kidney disease	*HNF1B*	Heterozygous deletion of whole gene
Structural variant	Distal myopathies	*PMP22*	Gain of whole copy of gene
Structural variant	Charcot-Marie-Tooth disease	*SETX*	CNV in compound heterozygosity with predicted damaging SNV
Structural variant	Inherited macular dystrophy	*BEST1*	Heterozygous deletion of exons 1-2 in compound heterozygosity with predicted damaging SNV
Structural variant	Rod-cone dystrophy	*PRPF31*	Heterozygous deletion of exons 4-12
Structural variant	Rod-cone dystrophy	*PRPF31*	Heterozygous deletion of exons 1-3
Structural variant	Rod-cone dystrophy	*PRPF31*	Heterozygous deletion of exon 1
Structural variant	Cone Dysfunction Syndrome	*CRX*	Homozygous deletion of whole gene
Structural variant	Rod-cone dystrophy	*CHM*	Heterozygous deletion in intron 12 with predicted cryptic splicing effect
Structural variant	Multiple bowel polyps	*APC*	Heterozygous deletion of exon 1

Structural variant	Stickler syndrome	*COL11A1*	Heterozygous deletion of exon 47
Structural variant	Congenital hearing impairment (profound/severe)	*PAX3*	Heterozygous deletion of exons 6-7
Structural variant	Dilated Cardiomyopathy (DCM)	*LMNA*	Heterozygous deletion of exon 5
Structural variant	Neuro-endocrine Tumours- PCC and PGL	*MAX*	Heterozygous deletion of exons 3-4
Structural variant	Epileptic encephalopathy	*WWOX*	Heterozygous deletion in intron 4 in compound heterozygosity with predicted damaging SNV
Structural variant	Intellectual disability	*AHDC1*	Heterozygous deletion of whole gene
Structural variant	Congenital hearing impairment (profound/severe)	*FOXC1*	Homozygous deletion of whole gene
Structural variant	Intellectual disability	*TCF4*	Heterozygous deletion of exon 1
Structural variant	Intellectual disability	*PRRT2*	Heterozygous deletion of whole gene
Structural variant	Bilateral microtia	*OTX2*	Gain of whole copy of gene
Structural variant	Hereditary spastic paraplegia	*SPAST*	Heterozygous deletion of exon 1
Structural variant	Hereditary spastic paraplegia	*SPAST*	Heterozygous deletion of exon 17
Structural variant	Congenital myopathy	*COL6A2*	Heterozygous deletion of exons 2-16
Structural variant	Hereditary spastic paraplegia	*SPAST*	Heterozygous deletion of exon 1

Table S8. Novel disease gene candidates.

22 novel candidates from the burden testing (with corresponding p values and overall false discovery rate q values adjusted for the total number of 590,451 tests) are shown, representing the most likely examples of fully penetrant Mendelian disease genes based on strict criteria of: (i) evidence from 3 or more unsolved, independent cases or 2 with existing functional evidence (either protein-protein interactions to known disease genes in the PanelApp disease panel coming from direct interactions with curated database or experimental evidence in StringDB[24] or from mouse models described by the International Mouse Phenotyping Consortium (IMPC)[25] or the Mouse Genome Database[26]), (ii) all variants driving the signal in the cases are completely absent from controls and absent from gnomAD or sufficiently rare given the expected prevalence, penetrance and age of onset of the disease, and (iii) observed/expected ratio of predicted LoF variants in gnomAD < 0.5 for predictions based on heterozygous, LoF variants

Disease	Gene	Predicted damaging variants in cases (heterozygous, in singleton cases and not observed in gnomAD unless otherwise stated)	Evidence
Hereditary spastic paraplegia	*UBAP1* (p=4.8x10^{-7}; q=0.002)	• ENST00000297661.4:c.535G>T:p.(Glu179*) in 4 cases<br>• ENST00000297661.8:c.373C>T:p.(Gln125*) in 1 case<br>• 3 cases involved larger family structures with affected siblings and affected and unaffected parents and in all cases the variant segregated with disease	• Only 3 LoF variants in controls, odds-ratio=65.7, and no nonsense variants<br>• GnomAD o/e LoF = 0.12 (0.05 - 0.38)<br>• Part of ESCRT-I/cargo complex with *VPS37A*, associated with spastic paraplegia type 53 and interacts with other spastic paraplegia genes: AP4E1, TUBB4A, AP4S1, KIF1A, ALS2, KIF5A, AP4M1 and AP4B1
Familial thoracic aortic aneurysm disease	*OPCML* (p=9.3x10^{-5}; q=0.078)	• ENST00000331898:c.752dup:p.(Met251Ilefs*3) in 1 case<br>• ENST00000331898:c.90del:p.(Pro30Profs*16) in proband but not unaffected parent in 1 case<br>• ENST00000331898:c.167+132134G>T::p.? splice donor variant in 1 case	• Only 2 LoF variants in controls, odds-ratio= 69.0<br>• GnomAD o/e LoF = 0.25 (0.12 - 0.57)
Ductal plate malformations	*SRP9* (p=1.5x10^{-5}; q=0.028)	• ENST00000304786.11:c.211C>T:p.(Arg71*) in 1 case<br>• ENST00000304786.11:c.3G>A:p.0? in 1 case	• Only 2 LoF variants in controls, odds-ratio= 632.4<br>• GnomAD o/e LoF = 0.19 (0.07 - 0.9)

			• Role in protein export pathway with other ductal plate malformation genes: *SEC61B* (polycystic liver disease 1) and SEC63 (polycystic liver disease 2)
Charcot-Marie tooth disease	*SORD* ($p=7.2x10^{-6}$; q=0.017)	• Hom ENST00000267814.9:c.757del:p.(Ala253Glnfs*27) in proband, het in unaffected parents in 1 case<br>• Hom ENST00000267814.9:c.757del:p.(Ala253Glnfs*27) in proband, het in unaffected parent in 1 case<br>• Het ENST00000267814.9:c.757del:p.(Ala253Glnfs*27) in compound heterozygosity with missense variants in 3 cases	• Variant never observed under a recessive MOI in controls and only one hom in gnomAD<br>• Only 2 control cases contain recessive, LoF variants in SORD<br>• *SORD* converts sorbitol to fructose and is part of the curated fructose and mannose metabolism pathway [KEGG] with *HK1*, associated with hereditary neuropathy (OMIM:605285)
Atypical haemolytic uraemic syndrome	*MAFG* ($p=3.1x10^{-5}$; q=0.044)	• ENST00000357736.4:c.395_397del:p.(Pro132del) disruptive inframe deletion in two cases	• Same variant in one periodic fever syndromes and amyloidosis control sharing some general genitourinary system abnormalities in common but no other LoF variants in controls (odds-ratio-374.7)<br>• GnomAD o/e LoF = 0 (0 - 0.69)<br>• Thrombocytopenia observed in patients and an IMPC mouse models with *Mafg* disruptions
Familial Thoracic Aortic Aneurysm Disease	*NRROS* ($p=3.8x10^{-5}$; q=0.050)	• ENST00000328557:c.190delC:p.(Leu64Trpfs*81) in two cases, 0.0004% in gnomAD<br>• ENST00000328557:c.346dupT:p.(Cys116Leufs*91) in proband and affected mother in 1 case	• Only 1 LoF variant in controls, odds-ratio= 138<br>• GnomAD o/e LoF = 0.23 (0.11 - 0.53)
Brain channelopathy	*KATNBL1* ($p=6.5x10^{-5}$; q=0.067)	• ENST00000256544:c.339delA:p.(Lys113Asnfs*51) in 2 cases, 0.0004% in gnomAD<br>• ENST00000256544:c.115_116insT: p.(Glu39Valfs*3) in 1 case	• Only 7 LoF variant in controls, odds-ratio= 51<br>• GnomAD o/e LoF = 0.17 (0.08 - 0.45)
Hereditary spastic paraplegia	*SLC35G2* ($p=2.4x10^{-5}$; q=0.037)	• ENST00000446465:c.896dup:p.(Ile300Asnfs*49) in 2 cases: in proband and affected sib in one of them<br>• ENST00000446465:c.290dup:p.(Asn97Lysfs*27) in 1 case, 0.0004% in gnomAD<br>• ENST00000446465:c.507_509del:p.(Phe170del) disruptive inframe deletion in 1 case, 0.0008% in gnomAD	• Only 4 LoF variant in controls, odds-ratio= 39<br>• GnomAD o/e LoF = 0.37 (0.18 - 0.85)

Primary immunodeficiency	*KDM4C* (p=1.4x10^{-4}; q=0.091)	• ENST00000420847:c.741+1G>A:p.? splice donor variant in 1 case, 0.002% in gnomAD<br>• ENST00000420847:c.196dup:p.(Cys66Leufs*15) in 1 case<br>• ENST00000536108:c.1060C>T:p.(Gln332*) in 1 case	• Only 13 LoF variant in controls, odds-ratio= 36<br>• GnomAD o/e LoF = 0.38 (0.27 - 0.54)<br>• Heterozygous *Kdm4c* null mice from IMPC exhibits immune phenotypes
Unexplained kidney failure in young people	*PLEKHD1* (p=5.1x10^{-5}; q=0.058)	• ENST00000322564:c.1015G>T:p.(Glu339*) in 1 case<br>• ENST00000322564:c.245G>T:p.(Gly82Val) in 1 case<br>• ENST00000322564:c.1159_1161del:p.(Glu387del) disruptive inframe deletion variant in 1 case	• Only 7 predicted damaging variants in controls, odds-ratio=55<br>• GnomAD o/e missense = 0.72 (0.65 - 0.81), o/e LoF = 0.47 (0.30 - 0.74)
Lipoedema disease	*CDH10* p=4.7x10^{-5}; q=0.054)	• ENST00000264463.8:c.2042T>C:p.(Ile681Thr) in 2 cases<br>• ENST00000264463.8:c.2173C>A:p.(Pro725Thr) in 1 case	• Only 18 predicted damaging variants in controls, odds-ratio=49<br>• GnomAD o/e missense = 0.69 (0.63 - 0.76), o/e LoF = 0.23 (0.14 – 0.42)
Extreme early-onset hypertension	*UBE2F* (p=7.9x10^{-5}; q=0.074)	• ENST00000272930.8:c.209C>T:p.(Thr70Ile) in 1 case, 0.0004% in gnomad<br>• ENST00000272930.8:c.53G>A:p.(Arg18Gln) in 1 case, 0.003% in gnomad<br>• ENST00000439338.5:c.149-6_149-5insA:p.? splice region variant in 1 case	• Only 9 predicted damaging variants in controls, odds-ratio=45<br>• GnomAD o/e missense = 0.64 (0.53 - 0.78), o/e LoF = 0.08 (0.03 - 0.38)<br>• Role in polyubiquitination of substrate pathway with other hypertension genes: CUL3 (Pseudohypoaldosteronism, type IIE) and KLHL3 (Pseudohypoaldosteronism, type IID)
Distal myopathies	*NSUN7* (p=1.2x10^{-4}; q=0.087)	• ENST00000381782.6:c.2028_2029del:p.(Tyr676fs) in 1 case, 0.003% in gnomAD<br>• ENST00000381782.2:c.1440_1441dup:p.(Ile481Lysfs*26) in 1 case, 0.003% in gnomAD<br>• ENST00000316607.5:c.1036+6T>C:p.? splice region variant in 1 case	• Only 9 predicted damaging variants in controls, odds-ratio=39<br>• GnomAD o/e LoF = 0.49 (0.32 – 0.79)
Non-CF bronchiectasis	*FOXJ1* (p=1.2x10^{-4}; q=0.088)	• *De novo* ENST00000322957.6:c.967del:p.(Glu323Serfs*10) in 2 trio cases<br>• ENST00000322957.6:c.1058T>G:p.(Ile353Ser)in 1 case, 0.002% in gnomAD	• Only 15 predicted damaging variants in controls, odds-ratio=36 and only 1 LoF variant<br>• GnomAD o/e LoF = 0 (0 - 0.28)<br>• Foxj1 KO mouse exhibits hydroencephaly and absent respiratory motile cilia modelling some patient phenotypes
Extreme early-onset hypertension	*FCHO1* p=1.6x10^{-4}; q=0.096)	• ENST00000593385.5:c.-14+8C>T:p.(=)splice region variant in 1 case<br>• ENST00000593385.5:c.-194-6C>G:p.(=)splice region variant in 1 case<br>• ENST00000593385.5:c.-14+5G>A:p.(=)splice region variant in 1 case	• Only 12 predicted damaging variants in controls, odds-ratio=34<br>• GnomAD o/e LoF = 0.14 (0.08 – 0.26)

			• Role in cargo recognition for clathrin-mediated endocytosis pathway with other hypertension genes: AGTR1 (essential hypertension)
Primary immunodeficiency	*ANKRD2* p=1.4x10^{-4}; q=0.091)	• ENST00000298808.9:c.566C>G:p.(Ser189Cys)in proband but not unaffected mother in 1 case<br>• ENST00000298808.9:c.541C>T:p.(Arg181Cys)in 1 case, 0.00006% in gnomAD<br>• ENST00000298808.9:c.283G>T:p.(Val95Leu)in 1 case, 0.0004% in gnomAD<br>• ENST00000307518.9:c.766C>T:p.(Arg256Trp) in 1 case, 0.006% in gnomAD	• Only 37 predicted damaging variants in controls, odds-ratio=17<br>• GnomAD o/e missense = 0.78 (0.69 - 0.89), o/e LoF = 0.79 (0.52 - 1.24
Mitochondrial disorders	*CITED2* (p=1.3x10^{-4}; q=0.089)	• ENST00000367651:c.685_686del:p.(Met229Valfs*25) in 1 case<br>• ENST00000367651:c.-7A>G:p.(=) splice region variant in 1 case<br>• ENST00000367651:c.559_585del:p.(Ala187_Gly195del) inframe deletion in 1 case, 0.00001% in gnomAD	• Only 6 predicted damaging variants in controls, odds-ratio=42 including 1 LoF<br>• GnomAD o/e LoF = 0 (0 - 0.63)
Lipoedema disease	*DIS3L2* (p=1.0x10^{-4}; q=0.082)	• ENST00000418143:c.254del:p.(Gly85Valfs*82) in 1 case, 0.009% in gnomAD<br>• ENST00000273009:c.491_514del:p.(Val164_Asp171del) inframe deletion in 1 case<br>• ENST00000424049:c.1148G>A:p.(Arg383Hisext*-383) stop lost variant in 1 case, 0.002% in gnomAD	• Only 24 predicted damaging variants in controls, odds-ratio=37<br>• GnomAD o/e LoF = 0.18 (0.1 - 0.33)
Rod-cone dystrophy	*RALGPS2* (p=5.6x10^{-5}; q=0.061)	• ENST00000367632.2:c.229C>T:p.(Gln77*) in proband but not unaffected parent. Observed at 0.003% in gnomAD<br>• ENST00000324778.5:c.462T>G:p.(Tyr154*) in 1 case<br>• ENST00000324778.5:c.-84+3C>T:p.(=) splice region variant in 2 cases	• Only 3 predicted damaging variants in controls, odds-ratio=35.3<br>• GnomAD o/e LoF = 0.23 (0.14 - 0.41)
Familial thoracic aortic aneurysm disease	*ABRAXAS2 (FAM175B)* p=2.4x10^{-5}; q=0.037)	• ENST00000298492.5:c.268-10_268-7del:p.? splice region variant in proband but not unaffected parent in 1 case<br>• ENST00000298492.5:c.578+3A>G:p.? spice region variant in 1 case<br>• ENST00000298492.5:c.689G>C:p.(Ser230Thr) in 1 case<br>• ENST00000298492.5:c.1013G>T:p.(Gly338Val) in 1 case	• Only 5 predicted damaging variants in controls, odds-ratio=82.3<br>• GnomAD o/e missense = 0.8 (0.71 - 0.9), o/e LoF = 0.32 (0.18 - 0.6)
Dilated cardiomyopathy	*BMP10* (p=7.6x10^{-5}; q=0.073)	• ENST00000295379.1:c.1263_1264del:p.(Cys421Trpfs*3) in 1 case<br>• ENST00000295379.1:c.953C>T:p.(Ala318Val) in proband and affected sibling in 1 case<br>• ENST00000295379.1:c.1037G>A:p.(Gly346Glu) in 1 case. 0.003% in gnomAD	• Only 4 predicted damaging variants in controls, odds-ratio=56.2<br>• GnomAD o/e missense = 0.81 (0.72 - 0.92), o/e LoF = 0.08 (0.03 - 0.39)<br>• Mouse knockout of *Bmp10* exhibits abnormal heart morphology, enlarged heart,

			haemorrhage, decreased heart rate and ventricular hypoplasia
Dilated cardiomyopathy	*PSMB11* (p=9.0x10^{-6}; q=0.020)	• ENST00000408907.2:c.169_171dup:p.(Phe57dup) inframe insertion in proband and affected mother but not unaffected father in 1 case<br>• ENST00000408907:c.499G>A:p.(Gly167Ser) in proband but not unaffected mother in 1 case, 0.002% in gnomAD<br>• ENST00000408907:c.502_503del::p.(Thr168Profs*48) in 1 case, 0.000004% in gnomAD	• Only 1 predicted damaging variant in controls, odds-ratio=224.4<br>• GnomAD o/e missense = 0.99 (0.88 - 1.11), o/e LoF = 1.14 (0.66 - 1.83)

Table S9. Reported healthcare benefits.

Disease	Gene	Change in medication	Additional surveillance for proband or relatives	Clinical trial eligibility	Informs reproductive choice	Other
Intellectual disability	*SLC2A1*	Y			Y	
Mitochondrial disorders	*FLAD1*	Y			Y	
Hereditary ataxia	*CACNA1A*	Y				
CAKUT	*RET*	Y	Y			
Rod-cone dystrophy	*USH2A*		Y	Y	Y	
Cone Dysfunction Syndrome	*CABP4*		Y	Y	Y	
Congenital hearing impairment (profound/severe)	*USH2A*		Y	Y	Y	
Rod-cone dystrophy	*USH2A*		Y	Y		
Rod-cone dystrophy	*BBS1*		Y	Y		
Inherited macular dystrophy	*BBS1*		Y	Y		
Rare multisystem ciliopathy disorders	*CEP290*		Y		Y	
Bilateral microtia	*SIX1*		Y		Y	
Bardet-Biedl Syndrome	*BBS1*		Y		Y	
Bardet-Biedl Syndrome	*BBS1*		Y		Y	
Rare multisystem ciliopathy disorders	*EVC*		Y		Y	
Intellectual disability	*PURA or RLIM*		Y		Y	
Familial exudative retinopathy	*LRP5*		Y		Y	
Hereditary spastic paraplegia	*SPAST*		Y		Y	
Hereditary spastic paraplegia	*CYP7B1*		Y		Y	
Hereditary ataxia	*SYNE1*		Y		Y	
Hereditary spastic paraplegia	*NF1*		Y		Y	
Inherited white matter disorders	*PSAP*		Y			
Brugada syndrome	*SCN5A*		Y			

Bardet-Biedl Syndrome	*BBS1*	Y			
Familial Tumours Syndromes of the central & peripheral Nervous system	*PTEN*	Y			
Long QT syndrome	*SLC16A1*	Y			
Bardet-Biedl Syndrome	*BBS1*	Y			
Dilated Cardiomyopathy (DCM)	*TTN*	Y			
Left Ventricular Noncompaction Cardiomyopathy	*MYH7*	Y			
Rod-cone dystrophy	*PROM1*		Y	Y	
Rod-cone dystrophy	*CERKL*		Y	Y	
Cone Dysfunction Syndrome	*ABCA4*		Y	Y	
Inherited macular dystrophy	*ABCA4*		Y	Y	
Rod-cone dystrophy	*AGBL5*		Y	Y	
Leber Congenital Amaurosis / Early-Onset Severe Retinal Dystrophy	*GUCY2D*		Y	Y	
Osteogenesis Imperfecta	*COL1A1*		Y		
Congenital hearing impairment (profound/severe)	*USH2A*			Y	Y
Intellectual disability	*SRD5A3*			Y	Y
Paediatric motor neuronopathies	*DYNC1H1*			Y	
Paediatric motor neuronopathies	*SPG7*			Y	
Early onset dystonia	*ADAR*			Y	
Hereditary spastic paraplegia	*KIF1A*			Y	
Rod-cone dystrophy	*ADAM9*			Y	
Hereditary spastic paraplegia	*CYP7B1*			Y	
Hereditary ataxia	*SPG7*			Y	
Congenital hearing impairment (profound/severe)	*MORC2*			Y	
Intellectual disability	*ALG11*			Y	
Rod-cone dystrophy	*IFT140*			Y	
Cone Dysfunction Syndrome	*KCNV2*			Y	
Stickler syndrome	*COL11A1*			Y	
Congenital hearing impairment (profound/severe)	*RAF1*			Y	

Intellectual disability	*ASPM*	Y	
Hereditary ataxia	*PLP1*	Y	
Rod Dysfunction Syndrome	*CACNA1F*	Y	
Intellectual disability	*KDM5B*	Y	
Mitochondrial disorders	*PNPT1*	Y	
Hereditary spastic paraplegia	*FA2H*	Y	
Ocular and oculo-cutaneous albinism	*OCA2*	Y	
Rod-cone dystrophy	*SNRNP200*	Y	
Intellectual disability	*SPG7*	Y	
Rod-cone dystrophy	*USH2A*	Y	
Mitochondrial disorders	*BOLA3*	Y	
Paediatric motor neuronopathies	*MFN2*	Y	
Inherited macular dystrophy	*BEST1*	Y	
Rod-cone dystrophy	*PRPF31*	Y	
Posterior segment abnormalities	*ABCA4*	Y	
Rod-cone dystrophy	*CRB1*	Y	
Rod-cone dystrophy	*FAM161A*	Y	
Inherited macular dystrophy	*ABCA4*	Y	
Cone Dysfunction Syndrome	*ABCA4*	Y	
Hereditary spastic paraplegia	*ZFYVE26*	Y	
Intellectual disability	*EXOSC3 or COQ2*	Y	
Congenital hearing impairment (profound/severe)	*SGSH*	Y	
Non-CF bronchiectasis	*SPAG1*		Y
Congenital hearing impairment (profound/severe)	*USH2A*		Y
Cone Dysfunction Syndrome	*CNGB3*		Y
Rod-cone dystrophy	*PRCD*		Y
Intellectual disability	*SLC2A1*		Y

Ocular and oculo-cutaneous albinism	*GPR143*	Y
Congenital hearing impairment (profound/severe)	*USH2A*	Y
Mitochondrial disorders	*PDHA1*	Y
Epileptic encephalopathy	*SMC1A*	Y
Corneal abnormalities	*SLC4A11*	Y
Rod-cone dystrophy	*USH2A*	Y
Anophthalmia/microphthamia	*BCOR*	Y
Intellectual disability	*MED13L*	Y
Rod Dysfunction Syndrome	*TRPM1*	Y
Rod-cone dystrophy	*RS1*	Y
Cataracts	*CRYBA1*	Y
Rod-cone dystrophy	*CNGB3*	Y
Cataracts	*RHO*	Y
Posterior segment abnormalities	*GUCY2D*	Y
Leber Congenital Amaurosis / Early-Onset Severe Retinal Dystrophy	*PRPF8*	Y
Ocular and oculo-cutaneous albinism	*TYR*	Y
Rod-cone dystrophy	*CRB1*	Y
Cataracts	*GJA3*	Y
Cataracts	*CRYAA*	Y
Cataracts	*GJA3*	Y
Charcot-Marie-Tooth disease	*BICD2*	Y
Rod-cone dystrophy	*PDE6B*	Y
Posterior segment abnormalities	*ABCA4*	Y
Posterior segment abnormalities	*ABCA4*	Y
Intellectual disability	*LRP2*	Y

Table S10. Diagnostic odyssey for participant with a *TCN2* diagnosis.

Treatment specialty	Inpatient		Outpatient		Emergency care		All secondary care	
	Admissions	Cost	Admissions	Cost	Admissions	Cost	Admissions	Cost
**Trauma and Orthopaedic Service**			1	£140			1	£140
**Paediatric Intensive Care Service**	2	£21,750					2	£21,750
**Paediatric Medical Oncology Service**	1	£12,531					1	£12,531
**Paediatric Service**	6	£42,121	1	£203			7	£42,324
**Neonatal Critical Care Service**	1	£2,143	1	£175			2	£2,318
**Well Baby Service**	1	£698					1	£698
**Emergency care**					2	£238	2	£238
**Total**	**11**	**£79,243**	**3**	**£518**	**2**	**£238**	**16**	**£79,999**

Table S11: Diagnostic odyssey for participant with a *CTPS1* diagnosis.

Treatment specialty	Inpatient		Outpatient		Emergency care		All secondary care	
	Admissions	Cost	Admissions	Admissions	Admissions	Cost	Admissions	Cost
**General Surgery Service**	1	£1,431	2	£286			3	£1,717
**Ear Nose and Throat Service**			2	£160			2	£160
**Paediatric Surgery**	3	£6,095	1	£196			4	£6,291
**Paediatric Clinical Haematology Service**	9	£177,841	18	£4,063			27	£181,904
**Paediatric Clinical Immunology and Allergy Service**	2	£1,257	16	£2,922			18	£4,179
**Paediatric Respiratory Medicine Service**			4	£569			4	£569
**Community Paediatric Service**			148	£35,991			148	£35,991
**Clinical Genetics Service**			3	£1,566			3	£1,566
**Paediatric Cardiology Service**			2	£474			2	£474
**Dermatology Service**	1	£1,068	4	£462			5	£1,530
**Paediatric Service**	30	£108,189	44	£8,188			74	£116,377
**Paediatric Neurology Service**	1	£3,536	3	£1,106			4	£4,642
**Dietetics Service**			11	£781			11	£781
**Emergency care**					2	£391	2	£391
**Total**	**47**	**£299,417**	**258**	**£56,763**	**2**	**£391**	**307**	**£356,571**

Table S12. Pilot diagnostic yield for NHS Genomic Medicine Service clinical indications (>10 pilot cases available).

Clinical indication name	100,000 Genomes Project recruited disease categories	Pilot cases	Diagnostic yield
Intellectual disability – microarray, fragile X and sequencing	Intellectual disability	132	40%
Hereditary ataxia with onset in adulthood	Hereditary ataxia with age at recruitment > 18yr	107	26%
Hereditary ataxia with onset in childhood	Hereditary ataxia with age at recruitment <= 18yr	13	38%
Early onset or syndromic epilepsy	Epileptic encephalopathy, Familial Genetic Generalised Epilepsies, Familial Focal Epilepsies	27	26%
Adult onset hereditary spastic paraplegia	Hereditary spastic paraplegia with age at recruitment > 18yr	64	39%
Skeletal dysplasia	Kyphoscoliotic Ehlers-Danlos syndrome , Multiple Epiphyseal Dysplasia, Thoracic dystrophies Unexplained skeletal dysplasia	18	6%
Cystic renal disease	Cystic kidney disease	27	22%
Bilateral congenital or childhood onset cataracts	Cataracts	23	48%
Adult onset neurodegenerative disorder	Amyotrophic lateral sclerosis/motor neuron disease, Complex Parkinsonism (includes pallido-pyramidal syndromes), Early onset and familial Parkinson's Disease, Early onset dementia (encompassing fronto-temporal dementia and prion disease)	31	20%
Congenital myopathy	Congenital myopathy	25	20%
Thoracic aortic aneurysm or dissection	Familial Thoracic Aortic Aneurysm Disease	30	10%
Primary immunodeficiency	A- or hypo-gammaglobulinaemia, Agranulocytosis, Combined B and T cell defect, Congenital neutropaenia , SCID	16	25%
Proteinuric renal disease	Proteinuric renal disease	11	46%
Bardet-Biedl syndrome	Bardet-Biedl syndrome	14	58%

Retinal disorders	Rod-cone dystrophy, Cone Dysfunction Syndrome, Inherited macular dystrophy, Leber Congenital Amaurosis / Early-Onset Severe Retinal Dystrophy, Rod Dysfunction Syndrome, Developmental macular and foveal dystrophy	196	46%
Structural eye disease	Ocular coloboma, Anophthalmia or microphthalmia, Glaucoma	16	33%
Non-syndromic hearing loss	Congenital hearing impairment (profound/severe), Autosomal dominant deafness	56	46%
Adult onset dystonia, chorea or related movement disorder	Early onset dystonia with age at recruitment > 18yr	25	24%
Paroxysmal central nervous system disorders	Brain channelopathy, Skeletal Muscle Channelopathies, Kleine-Levin syndrome	28	7%
Congenital malformation and dysmorphism syndromes	Bilateral microtia, Choanal atresia, Ear malformations, Familial hemifacial microsomia, Familial non-syndromic clefting, Kabuki syndrome, Paediatric congenital malformation-dysmorphism-tumour syndromes, Rare multisystem ciliopathy disorders, RASopathies, Sotos syndrome	24	25%
Hereditary neuropathy or pain disorder – NOT PMP22 copy number	Charcot-Marie-Tooth disease, Paediatric motor neuronopathies	101	35%
Other rare neuromuscular disorders	Distal myopathies, Rhabdomyolysis and metabolic muscle disorders	19	16%
Possible mitochondrial disorder - nuclear genes	Mitochondrial disorders	76	40%
